# STDN-GEN: rapid synthesis of layered critical-material dependency networks for supply-chain sustainability analysis

**DOI:** 10.21203/rs.3.rs-10767641/v1

**Published:** 2026-09-10

**Authors:** Aaron Schroeder, Mandy L. Wilson, Galen S. Harrison, Dustin Machi, Brian Klahn, Samarth Swarup, Anil Vullikanti, Achla Marathe, Margaret Foster, Philip B.K. Potter, Madhav V. Marathe

**Affiliations:** 1* Biocomplexity Institute, University of Virginia, Charlottesville, VA, USA.; 2 National Security Data and Policy Institute, University of Virginia, Charlottesville, VA, USA.

**Keywords:** critical materials, supply chain, multi-agent systems, large language models, sustainability, dependency networks

## Abstract

Assessing a critical-material supply chain first requires a map: which components make up a technology, which materials those components need, and which countries produce those materials. Building such maps is slow, manual work, which limits how many technologies an analyst can screen. We introduce STDN-GEN, a system in which several large-language-model agents draft these maps and reconcile their proposals against a curated vocabulary of component and material names. The output is an auditable four-level network from technology to component to material to producing country, built in minutes rather than days. Across 180 technologies in microelectronics, biotechnology, and pharmaceuticals, enforcing the shared vocabulary is by far the largest source of run-to-run reproducibility, raising agreement between repeated analyses of the same technology roughly fivefold; debate between agents helps in some domains and widens the set of candidate dependencies in others. The system recovers 98% of the components human annotators identified, and its maps reproduce the extreme production concentration that published assessments report for neodymium.

## 1 Introduction

The materials behind modern manufactured technologies are produced in a small number of countries, refined through multi-tier supply chains, and exposed to geopolitical, environmental, and logistical shocks.

The dependence cuts across sectors: semiconductor fabrication, medical imaging equipment, communications infrastructure, industrial robotics, and pharmaceutical and bioprocessing equipment (among many others) all rely on materials whose supply is geographically concentrated [1, 2]. The 2020–2022 semiconductor shortage showed how the loss of a few upstream inputs, such as specialty process chemicals, noble gases, and a handful of logic-node foundries, can cascade into years-long disruptions across otherwise unrelated industries [3].

Assessing this exposure for even one technology means tracing it through its components, the materials those components require, and the countries that produce those materials. Building a single such map is slow, manual work: analysts stitch together USGS commodity reports [1], material-flow assessments, and product teardowns in a fragmented, analyst-dependent process that runs to days per technology and still leaves coverage gaps [4, 5]. These limitations constrain how many technologies can be screened. At the same time, geopolitical realities are increasing the number of sectors and products that require assessment. STDN-GEN removes the bottleneck: it constructs auditable, layered dependency maps in minutes rather than days, at the speed and scale that portfolio-wide screening requires.

Criticality assessment has produced many methods, but they share a constraint. Methods range from multi-dimensional supply-risk scorecards [6, 7] and sector-specific supply-risk metrics [8] to resilience indicators that quantify recovery after shocks such as the 2010 rare-earth crisis [9], and analyses of the labor and justice dimensions of concentrated production [10]. What they hold in common is that the technology-to-material-to-country linkages they depend on must still be assembled by hand. Automation has addressed only parts of this. Input-output tables provide economy-wide material accounts [11], but they aggregate across industries rather than resolving an individual technology or the component-level decomposition a product engineer or policy analyst needs. Material-flow analysis and life-cycle assessment reach product-level granularity, yet building and maintaining per-technology inventories is labor-intensive and rarely reaches the sub-component tier. Substitution studies for rare earths in wind turbines show both the value and the cost of that work [12, 13]. Large language models (LLMs) have begun to help with materials-science extraction [14–16], alongside domain-adapted models such as MatSciBERT [17] and rule-based toolkits such as ChemDataExtractor [18]; knowledge-graph methods have been applied to critical-mineral analysis, including LLM-assisted retrieval over USGS commodity data [19], and multi-agent debate has improved reliability on factual and reasoning tasks [20]. None of these, however, produces an explicitly layered, auditable representation that traces a product from components to materials to producing countries, or validates its output against a structured domain ontology. This paper addresses that gap with a system fast enough for broad screening, layered enough for tier-specific analysis, and auditable enough to support applications such as concentration indices and stress tests [4].

We define the shallow technology dependency network (STDN) as a layered directed acyclic graph (DAG) that links a target technology to (i) its major manufacturing components, (ii) the materials those components use, and (iii) the top-producing countries for each material. We call it shallow because its depth is bounded at these four tiers, with no further sub-component or process-step decomposition. That bound is what makes construction fast and scalable, and the four tiers still capture the multi-tier coupling that drives supply vulnerability. We introduce **STDN-GEN**, a multi-agent framework that constructs STDNs from unstructured technology descriptions. It rests on three design elements: specialized LLM-based extraction agents operating at each tier; agreement-feedback-driven multi-agent debate for component extraction, which improves coverage through iterative peer critique and convergence scoring; and ontology-backed canonical normalization, which maps extracted terms onto a stable shared vocabulary. The output is an auditable layered DAG whose nodes are grounded in canonical terms, so results stay comparable across technologies and across repeated runs.

We evaluate STDN-GEN on 180 technologies across three manufacturing domains: 60 in microelectronics, 60 in biotechnology, and 60 in pharmaceuticals. Throughout, *N* denotes the number of component-extraction agents taking part in the debate, so *N*=1 is a single agent with no debate and *N* =3 is a three-agent debate. The layer-wise ablation (Sections 2.2 and 2.5, including the *N*=1 through *N*=5 cost–convergence sweep) uses microelectronics only; the cross-domain debate-effect comparison (Section 2.3) spans all three domains at the *N*=1 versus *N*=3 contrast.

Because the pipeline is stochastic, the same technology submitted twice need not return the same component list, and an analyst cannot build on a map that changes each time it is regenerated. We therefore run every technology five times under each configuration and ask how far the repeated runs agree. We call that agreement stability and measure it as the Jaccard similarity (size of intersection over size of union) between the component sets returned by each pair of runs on the same technology, taking the median over pairs: a system that returns the same components every time scores 1, one whose runs share nothing scores 0. The ablation then isolates where that stability comes from: a naive single-shot baseline (one unstructured prompt, with no role conditioning, no output schema, and no canonical vocabulary, standing in for what an analyst gets by asking an LLM the question directly), canonical normalization alone, structured extraction without debate, and the full pipeline with *N*-agent debate.

The headline result is that canonical normalization is the dominant lever for stability, while multi-agent debate’s contribution to post-normalization stability is domain-dependent and significant in opposite directions across the three domains studied. Mapping the naive baseline’s component names onto the canonical vocabulary, without touching the prompt or the model, raises median pairwise Jaccard from 0.146 to 0.756. Debate then lowers stability in microelectronics and raises it in biotechnology and pharmaceuticals; each per-domain effect is significant under a paired Wilcoxon signed-rank test, but because the effects point in opposite directions, they offset one another when the domains are pooled. An analysis of the pooled sample alone would have reported no effect of debate at all and missed the domain-specific structure entirely. We validate the pipeline with a gold-standard protocol on four technologies (recall 0.981 on a 53-component reference set) and complement the large-scale results with a smartphone case study, a collaborative robot arm vignette, and a 5G base-station vignette, each showing how layered STDNs surface processing-tier concentration bottlenecks, for example in gallium and indium, that component-only analyses miss.

By cutting the time to a first dependency map from days to minutes, STDN-GEN makes it feasible to screen the critical-material portfolios that frameworks such as the IEA Critical Minerals Outlook [4] and the EU Critical Raw Materials Act [5] track, rather than a handful of technologies at a time. Because the outputs are canonical and judge-validated, they feed directly into downstream quantitative work: Herfindahl–Hirschman Index (HHI) concentration scoring, in which a market’s concentration is summarized by the sum of squared producer shares, alongside stress-test scenarios and cross-technology coupling analysis. The same pairing of structured ontology grounding with multi-agent exploration extends to other automated sustainability-extraction problems.

## 2 Results

### 2.1 Layered STDNs surface processing-tier concentration bottlenecks

Layered STDNs reveal concentration risks that component-only analyses miss, because concentration can arise at the refining stage even when upstream extraction is geographically dispersed.

The representation is organized into four tiers, its nodes partitioned into four disjoint sets: technologies *T*, components *C*, materials *M*, and producer countries *P*. Each is a set rather than a single node, and lowercase letters denote members: a technology *t* ∈ *T* decomposes into a subset of *C*, each component *c* ∈ *C* draws on a subset of *M*, and each material *m* ∈ *M* is produced by a subset of *P*. Edges are constrained to adjacent tiers, producing a directed acyclic graph (DAG) compact enough to be generated automatically and inspected by an analyst in minutes. Per-edge confidence and provenance labels let analyst judgments be conditioned on data quality: *M* → *P* edges backed by USGS commodity statistics are authoritative, while model-inferred edges are flagged for targeted validation. Figure 1 shows the pipeline that synthesizes this representation. Each of the three extraction stages runs a propose–critique–revise loop: agents propose candidates independently, a deterministic agreement-feedback step labels each candidate as consensus, ma jority, or isolated, and agents revise against that feedback until the normalized proposals converge or a round cap is reached; ontology-backed canonical normalization then converts the result into auditable STDN edges. Figure 2 instantiates the representation for the smartphone, which combines roughly 15 primary components with critical materials whose production is geographically concentrated; node size encodes Herfindahl–Hirschman Index (HHI) concentration. The layered view surfaces what a component-only analysis cannot: indium, used in the transparent conductive coatings central to touchscreen and display functionality [21], is recovered from zinc deposits mined across many countries, yet refining capacity is concentrated in a few jurisdictions (China 65.7%, Korea 20.2%). Tantalum shows the same pattern, mined across Africa and Australia but processed under concentrated conditions, and cobalt from battery cathodes is dominated by the Democratic Republic of Congo at the mining stage. The *M* → *P* tier is the only place these processing-layer choke-points become visible.

Two vignettes (Figure 3) show the same pattern in other technology classes. Panel A shows a collaborative robot (cobot) arm, whose network exposes a rare-earth bottleneck: both the Actuation and Mounting System and the Sensors and Instrumentation components depend on neodymium, the element at the core of the NdFeB permanent magnets used in servo motors and encoder assemblies. China accounts for 75% of neodymium production (United States 10%, Australia 7%), a concentration sitting entirely at the refining tier, since neodymium ore occurs across multiple continents but processing capacity is narrow. Panel B shows a 5G base station macro cell, whose radio unit surfaces gallium arsenide, the compound semiconductor central to the radio-frequency front end, produced 75% in China (Germany 10%, Japan 7%), while the antenna system independently flags ceramics as 60% concentrated in China. Across consumer electronics, industrial robotics, and digital infrastructure, then, processingtier concentration is a general structural feature of layered STDNs rather than an artifact of one technology class, recurring across distinct material classes and sectors.

Such maps take under an hour per technology rather than days of desk research; Section 3 sets out what this makes tractable. The next two subsections measure how much of the output quality is attributable to normalization versus debate.

### 2.2 Ontology-backed normalization is the dominant lever for run-to-run stability

STDN-GEN is stochastic: the same technology submitted twice will not necessarily return the same component list, and an analyst cannot build on a map that changes each time it is regenerated. We quantify run-to-run stability by running every technology five times under a fixed configuration and comparing the resulting component sets pairwise. For two runs producing sets *A* and *B*, the Jaccard similarity |*A* ∩ *B*|/|*A* ∪ *B*| is the fraction of all components named by either run that both agree on: 1 when the runs are identical, 0 when they share nothing. Per technology we take the median over the $\left(\begin{array}{c} 5\\ 2 \end{array}\right)=10$ run pairs; per configuration, the median of those. Unless stated otherwise, sets are compared after canonical normalization, so two runs naming the same component differently count as agreeing.

Canonical normalization produces a roughly 4–5× improvement in median pairwise Jaccard at no additional inference cost.

The evidence appears in Table 1 and Figure 5A. The naive single-shot prompt yields a median pairwise Jaccard of 0.146 (95% CI 0.12–0.18) across 60 microelectronic technologies, at a median of 39.5 unique components per run. The canonical vocabulary is not hand-authored in advance. It is a persistent store of surface-form-to-canonical-name mappings that starts empty and accumulates across runs: a rule-based pass collapses trivial variants (case, punctuation, generic qualifiers such as “module”), and surviving variants go to an LLM that assigns each to an existing canonical name or opens a new one. Mappings are written back and reused, so only unseen variants incur a call (Section 4.4). Applying this vocabulary to that same raw output, without any change to the extraction prompt or the model, raises median Jaccard to 0.756 (95% CI 0.70–0.80), a gain of +0.61. Median component count falls from 39.5 to 12.0, because variants the naive output treats as distinct (e.g., “DRAM”, “Dynamic Random Access Memory”, “DRAM chip”) collapse onto one canonical name. Structured extraction, the next rung in the ablation ladder, further reduces component count and makes the invalid rate measurable. The invalid rate, our scalable precision proxy, is the fraction of extracted components that an LLM judge labels as not a real, in-scope manufacturing component for the technology, whether hallucinated, too generic, or belonging to another tier (Section 4.5). It is only meaningful once extraction emits a structured, typed list: the naive baseline’s free text mixes components with materials, processes, and prose fragments, leaving no well-defined set to adjudicate. The full pipeline at *N*=3 is characterized in §2.3.

Normalization dominates because surface-form variation is the single largest source of run-to-run disagreement in naive LLM output. Two runs that identify the same physical component often express it differently, and Jaccard computed on raw strings counts these as mismatches; canonical mapping absorbs that variation however it arises. Structured extraction, by contrast, uses role-conditioned prompts with explicit exclusion rules that reduce surface-form variation at the source, so the marginal benefit of normalization on top of structured extraction is smaller than its benefit on raw output. The diminishing-returns pattern is visible across the ablation rungs in Figure 5A: the largest single jump is always the canonical vocabulary, not any change to the extraction or debate mechanism. A light-prompt-engineered variant of the naive baseline (role conditioning, JSON output, one-sentence exclusion rule; Supplementary A) does not narrow the gap: raw stability is statistically indistinguishable from the strawman prompt at *n*=4 (0.179 versus 0.182), and the normalization gain is essentially unchanged at roughly +0.63. The finding is therefore not an artifact of a maximally weak baseline.

The result also generalizes across domains. In the cross-domain pilot (Section 2.3), the structured pipeline’s post-normalization median Jaccard at *N*=1 sits well above the naive baseline of 0.146 in every domain: 0.500 in microelectronics, 0.300 in biotechnology, 0.333 in pharmaceuticals, confirming that the same vocabulary infrastructure does substantive work outside the microelectronics setting of Table 1. None of this makes multi-agent debate redundant. Debate’s contribution is simply not a single direction of effect: in some domains it broadens the explored space, in others it stabilizes the canonical output, as the next subsection characterizes.

### 2.3 Multi-agent debate has a domain-dependent effect on output stability

Multi-agent debate’s effect on post-normalization stability is domain-dependent: it lowers stability in microelectronics and raises it in biotechnology and pharmaceuticals. The combined-sample null (Table 2, bottom row) arises because the per-domain effects run in opposite directions, not because no effect exists.

Table 2 and Figure 4 report the paired comparison on 177 technologies (60 microelectronics, 59 biotechnology, 58 pharmaceuticals; three dropped for insufficient paired-replicate runs). Debate moves the per-technology median post-normalization Jaccard down in microelectronics (Δ = −0.173, 49 of 60 technologies worsening, *p* < 0.0001) and up in biotechnology (Δ = +0.107, *p* = 0.0056) and pharmaceuticals (Δ = +0.036, *p* = 0.0092). Pooled, the combined median difference is −0.031 with paired Wilcoxon *p* = 0.55 and a bootstrap 95% CI of (−0.0359, +0.0333) straddling zero: the per-domain effects cancel.

Why the effect splits this way is an open question. The obvious candidate was that each domain’s component-vocabulary structure drives the sign, so we tested three pertechnology metrics on the 177 paired technologies: canonical-component cardinality, raw-name cardinality, and average synonym-set size (Supplementary A). Spearman correlations with the paired Δ are weak (|*ρ*| ≤ 0.13 pooled, all 95% bootstrap CIs straddling zero, all *p* > 0.09), and the per-domain distributions are close (canonical-cardinality medians 18, 21, 20; raw-cardinality medians 52.5, 54.0, 53.5). A simple vocabulary-cardinality mechanism therefore does not explain the split. Because the vocabulary grew during the pilot, we also re-normalized all 60 cross-domain raw CSVs against a frozen post-pilot vocabulary and recomputed Table 2: the per-domain *p*-values and Δ values are unchanged (Supplementary A).

Where debate lowers post-normalization Jaccard, in microelectronics, the same broadening doubles as a coverage feature for analysts willing to triage a wider candidate set: the core ratio at *N*=3 is 0.200 versus 0.429 at *N*=1. Debate strength is thus not a single transferable hyperparameter, a point Section 3 develops into deployment guidance.

### 2.4 Gold-standard validation confirms judge reliability

The LLM judge used in §2.2 and §2.3 to estimate component validity at scale closely matches multi-annotator gold-standard lists.

Across four technologies spanning pharmaceutical and biotech manufacturing equipment and consumer electronics, covering 53 canonical components (Table 3), it achieves aggregate recall 0.981 and lower-bound precision 0.722. The recall figure means the judge identified as plausible 52 of the 53 gold-standard components; the single false negative is “Connectors” on the Smartphone, which it classified as secondary rather than primary. The precision bound is deliberately conservative, treating every system-extracted component absent from the gold standard as a false positive. Manual inspection of these 20 apparent false positives shows most are legitimate primary components the annotators omitted, for example Door Assembly and Condenser/Cold Trap System for lyophilizers, and Lithium-ion Battery Module and PCB Assembly for the Smartphone. Re-adjudication therefore suggests substantially higher true precision, though with *n*=4 technologies we bound the claim to this sample. The annotation protocol, a multi-annotator enumeration merged by consensus adjudication and canonicalized before comparison, is described in Section 4.6; only Smartphone overlaps the 60-technology microelectronics benchmark, so the other three extend validation beyond it.

The judge-based precision proxies in §2.2 and §2.3 should therefore be read as conservative: true precision is at least as good as those figures suggest, and likely better, given the systematic gap between the gold-standard count and the full universe of legitimate components.

### 2.5 Cost and convergence as a function of debate strength

Cost grows roughly linearly with agent count while the marginal quality benefit shrinks, motivating a practical sweet spot near *N*=3 for batch analysis.

Across the 60-technology microelectronics benchmark, median per-technology runtime rises from 10.6 minutes at *N*=1 through 23.4, 32.3, and 42.2 minutes to 49.5 minutes at *N*=5, roughly 5× over the sweep. Debate rounds grow from 1.00 to 2.20 over the same range, so stronger debates trigger more revision cycles, yet macromedian final convergence (Jaccard overlap across agent proposals in the last round) barely moves, from 0.930 at *N*=2 to 0.894 at *N*=5; with no peer to converge with, it is undefined at *N*=1.

Quality gains show the same flattening. Moving from *N*=1 to *N*=3 captures most of the transcript-level stability gain, roughly doubling pre-normalization Jaccard from 0.073 to 0.147 with non-overlapping 95% CIs (0.055, 0.089) versus (0.123, 0.166) (Figure 5B), and shifts the median occurrence-based invalid rate from 0.011 to 0.000, though that improvement is not systematic per technology (paired Wilcoxon *W* =240, *p*=0.33; 17/60 improved, 17/60 worsened, 26/60 tied). Gains from *N*=3 to *N*=5 are incremental while runtime keeps climbing at nearly the same rate per agent. In practice, 50 technologies at *N*=3 complete in roughly 27 hours on a single process against 41 hours at *N*=5. Larger *N* remains justified for a high-priority technology where breadth matters more than throughput and a domain expert will triage the output.

API cost is negligible by comparison. At gpt-4.1-mini pricing (input $0.15 per 1M tokens, output $0.60 per 1M tokens, as of the run dates), per-technology cost on a 5-technology held-out subset averages $0.0019 at *N*=1 and $0.0041 at *N*=5; gpt-4.1 normalization and judge calls add an estimated $0.0005 and are excluded from the primary figure. These cover Stage 1 and Stage 2 only, since Stage 3 country data is served from the USGS database and a persistent fallback cache. Supplementary Figures 11 and 12 give the full sweep, including substantial per-technology runtime variance (complex component spaces trigger more rounds at the same *N*) and Stage 2 materials debate, where stronger debate yields more consistent but shorter materials lists.

### 2.6 Downstream sustainability analysis: concentration indices for a CRM-listed material across the benchmark

Consider a concrete analyst question: which technologies in a portfolio share a single-country dependency on a critical material? Answering it traditionally takes weeks of desk research per material, cross-referencing criticality databases against component-level documentation and rolling up dependencies by hand. With layered STDN outputs in place, it reduces to a one-line aggregation over the country-tier table with auditable provenance. We work the example for neodymium, both to demonstrate the path and to validate STDN-GEN’s country-tier outputs against the published criticality literature.

Neodymium is a light rare-earth element on the EU 2024 Critical Raw Materials list [5] and the USGS 2022 critical-minerals list. Taking the union across five repeated *N* =5 runs, it surfaces in seven benchmark technologies spanning consumer electronics (Smart Speaker, Smart Doorbell, Laptop PC, Large-Format OLED Television, Hearing Aid) and industrial robotics (Collaborative Robot Arm, Servo Motor Drive), in each case as the core of NdFeB permanent magnets in audio transducers, servo motors, and small actuators. The country distribution recovered is the same for every one (China 75%, United States 10%, Australia 7%, Russia 5%, India 3%), giving a per-technology HHI of 75^2^+10^2^+7^2^+5^2^+3^2^ = 5,808, more than double the US DOJ/FTC “highly concentrated” threshold of 2,500 (Figure 6).

The published literature treats neodymium as a paradigmatic concentrated supply case [6, 8]: the USGS 2022 list flags it for high import reliance, and the IEA reports China at roughly 60% of mined rare-earth production and more than 90% of refined supply in 2023 [4]. STDN-GEN lands in the same regime from independent extraction, without consulting any published criticality assessment, at roughly 32 minutes per technology at *N*=3 (§2.5) against the weeks that comparable manual work requires. It also resolves the verdict per technology rather than only per material, which is what surfaces the portfolio-level finding: the concentration verdict is invariant to the downstream technology, because industrial automation and consumer electronics draw their neodymium from the same concentrated processing base. Mitigations such as refining-capacity diversification, stockpiling, or recycling mandates therefore act on one chokepoint propagating across sectors simultaneously. The same disaggregation can be produced for any CRM-listed material in the runtime of a single run; Section A reports full CRM coverage across the benchmark, with caveats for materials not surfaced and for technology classes (photovoltaics, wind, batteries) that would broaden it further.

## 3 Discussion

STDN-GEN makes layered dependency analyses tractable at the speed and scale that time-constrained policy work requires. Concrete decision contexts where this matters include the triennial USGS critical-minerals list update (published within the annual Mineral Commodity Summaries), the EU Critical Raw Materials Act’s biennial review of strategic and critical raw materials, the IEA Critical Minerals Outlook’s technology-portfolio assessments, and analogous vulnerability reviews by national-security, energy, and industrial-strategy agencies.

The smartphone case study and the collaborative robot arm and 5G base station vignettes (Figure 2 and Figure 3) illustrate the kinds of analyses that become possible. STDN-GEN enables rapid vulnerability screening across technology portfolios; comparative critical-material analysis across sectors, for example contrasting which industrial-robotics and energy-transition technologies share rare-earth refining bottlenecks; and cross-technology bottleneck identification, for example finding which processing-tier suppliers serve multiple strategic products simultaneously. Section 2.6 carries out one such analysis end-to-end: per-technology Herfindahl–Hirschman Index for Neodymium across the seven benchmark technologies that surface it, with concentration verdicts consistent with USGS and IEA published criticality assessments. Each of these analyses is currently expensive to produce manually for even a single technology. Automated layered synthesis collapses the cost per technology to under an hour (Section 2.5), making batches of dozens of technologies tractable within a working day.

The auditable structure (canonical vocabulary plus judge-validated outputs) supports downstream uses including concentration indices (HHI-based or analogous), comparative disruption stress tests, and longitudinal monitoring as supply chains reconfigure in response to policy, investment, or geopolitical pressure. Concretely, this makes it feasible to refresh a portfolio’s STDN snapshot whenever the policy basis changes (a new critical-materials list, an export-control announcement, a refinery out-age) and re-run downstream concentration and stress-test analyses on the updated maps within the same working day, shifting dependency mapping from a quarterly artifact toward continuous infrastructure for sustainability decision-making.

Shallowness is a design choice. STDNs surface concentration at the producing-country layer for each constituent material, but they do not decompose that layer further into mining-versus-refining splits or specific facility-level dependencies, and they report producer concentration in aggregate (top-*N* countries by share) rather than as full geographic distributions. The choice trades resolution for synthesis speed and auditability, and it reflects what the available public sources reliably support: USGS commodity reports, BACI/Comtrade trade flows, and similar open databases. The representation is appropriate for first-pass vulnerability screening and comparative analysis across technologies. It is not appropriate for design-level supply chain optimization, supplier-specific risk underwriting, or detailed financial exposure modeling, which require resolution that STDNs do not provide. The Stage 2b extraction of process consumables (e.g., specialty gases, etchants) partially addresses the omission of manufacturing-process inputs, but intermediate processing steps, sub-assembly hierarchies, and temporal dynamics remain outside the representation. Variable-depth extensions (deeper decomposition where warranted by strategic importance, shallower elsewhere) and time-indexed STDN snapshots that track supply-chain reconfiguration over years are natural next steps. Integration with explicit risk-scoring modules such as Herfindahl-Hirschman indices, country-risk indices, and geopolitical scenario stress tests would let STDN outputs feed standard sustainability and resilience analyses [22, 23] without manual reshaping. Finally, the LLM judge used to estimate validity at scale is itself an extraction system trained on the same kinds of unstructured sources as the extractor; cross-source validation reconciling LLM-extracted material lists against USGS commodity reports is partially implemented and warrants strengthening.

Three caveats apply to any use of STDN outputs. First, outputs are bounded by the availability, timeliness, and accessibility of underlying public sources. Proprietary supply relationships and weakly documented technologies (often the most strategically interesting cases) may be missing or ambiguous in STDN output, and the system has no mechanism for flagging known-unknowns. Second, judge-based validity is a useful scalable signal but does not capture correlated errors or systematic failure modes. A judge trained on the same kinds of sources as the extractor may share blind spots; the gold-standard validation (Table 3) bounds this concern for the four annotated technologies but does not extrapolate to all 60 in the benchmark. Calibration analyses, error taxonomies, and benchmarked evaluations against independent ground truth are open work. Third, the canonical vocabulary that drives normalization is built from the same source ecosystem as the extracted content, and therefore inherits whatever taxonomic biases that ecosystem carries. The current implementation is English-centric, which restricts coverage of supply-chain literature published in Mandarin, Japanese, Korean, German, and other languages that document large fractions of global manufacturing activity. This restriction is consequential for sustainability applications: key materials in energy-transition and semiconductor supply chains are processed and traded by firms whose documentation is published primarily in non-English languages. The vocabulary also grew during the pilot, but pre-existing coverage of incoming raw component names was similar across domains (12.2 to 14.7%) and a frozen-vocabulary re-normalization leaves the cross-domain statistics unchanged (Supplementary A). Broadening the source-language base, improving cross-source validation against trade and customs data, and developing calibration and error taxonomies are concrete next steps that would strengthen STDN-GEN’s utility for sustainability-focused supply-chain analysis.

A note on domain-dependent debate behavior. The cross-domain result in Section 2.3 shows that debate’s sign of effect on post-normalization stability differs by domain (debate lowers Jaccard in microelectronics, raises it in biotechnology and pharmaceuticals), and the effect is statistically significant in all three cases. The mechanism that produces this split is an open question. We tested whether per-technology canonical-component cardinality, raw-name cardinality, or average synonym-set size predicts the per-technology paired delta and found weak Spearman correlations within and across domains, with the qualitative ordering of the three domains on these metrics being roughly equivalent rather than predictive (Supplementary A). We therefore cannot recommend a single measurable diagnostic that tells a practitioner in advance whether debate will help or hurt in a new domain. The deployment guidance that remains supported by the data is empirical calibration: when deploying STDN-GEN in a new domain, run a small pilot at multiple debate strengths and compare paired stability before fixing a default.

A note on dual-use considerations. Large-scale, layered supply-chain dependency mapping is intelligence-relevant: the same tools that enable rapid sustainability vulnerability screening can in principle support targeting of supply-chain chokepoints. STDN-GEN surfaces only information that is already public (USGS commodity reports, EU CRM Act lists, BACI/Comtrade trade flows, technical product documentation); the system has no access to proprietary, classified, or otherwise restricted data sources. The pipeline’s contribution is synthesis speed and structured-output auditability, not novel intelligence. We release the code openly to ensure that this capability is broadly accessible to sustainability researchers, policy analysts, and civil society organizations rather than concentrated in actors with privileged access.

During vignette construction we observed that the NdFeB rare-earth magnet dependency in a collaborative robot arm (concentrated 75% in China) surfaced only at *N*=5. We treat this as a vignette-level observation rather than a systematic property of the debate mechanism; whether such depth gains generalize across the benchmark is an open question for future work.

### 3.1 A design lesson for automated sustainability-knowledge extraction

For teams building automated tools for analogous supply-chain or LCA-style knowledge tasks under a fixed engineering budget, our results carry a practical message: canonical-vocabulary investment should come before multi-agent ensembling, and the appropriate debate strength after that investment is best determined by an empirical pilot in the target domain. We observed a roughly 4–5× stability improvement from canonical normalization alone (Table 1; conservative 95% bootstrap CI ratio ≈ 3.9×, median ratio ≈ 5×). Adding debate on top of structured extraction produces a domain-dependent effect on post-normalization stability: a significant decrease in microelectronics (Δ = −0.173, *p* < 0.0001), and significant increases in biotechnology (Δ = +0.107, *p* = 0.0056) and pharmaceuticals (Δ = +0.036, *p* = 0.0092) (Table 2). Normalization absorbs surface-form variance, which is the dominant source of run-to-run disagreement in LLM-extracted content. Debate’s effect is more complicated: we attempted to attribute it to per-technology vocabulary cardinality but found no supporting correlation (Supplementary A), so we cannot offer a measurable a-priori predictor for whether debate will help or hurt in a new domain. Teams beginning a new extraction pipeline frequently invest first in prompt engineering and multi-agent ensembling because those are immediately actionable; they should consider that an upstream ontology investment (even a partial canonical vocabulary covering the most common naming variants) may yield larger returns before those secondary refinements are added, and that debate’s downstream contribution should be calibrated empirically in each target domain rather than transferred across domains.

The lesson generalizes beyond supply-chain analysis. Sustainability-knowledge tasks (lifecycle inventories, criticality assessments, material flow models, climate-impact accounting) share the same surface-form variance problem: the same material or component appears under many names, and set-based agreement metrics collapse until that variance is absorbed. Domain ontologies should be treated as first-class engineering artifacts in such systems, not as afterthought post-processing. Multi-agent ensembling complements vocabulary investment; it is not a substitute for it.

## 4 Methods

An STDN is a layered directed acyclic graph G(V,E) where the vertex set comprises four disjoint layers: technologies T, components C, materials M, and producer countries P. Edges are constrained to adjacent layers: $E\subset T\times C\cup C\times M\cup T\times M_{\text{proc}}\cup M\times P$, where $M_{\text{proc}}\subset M$ denotes process consumable materials consumed during manufacturing but absent from the final product (e.g., process gases, etchants, solvents). Process consumables connect directly to the technology layer, bypassing the component layer, because they are associated with the manufacturing process as a whole rather than individual components. Each edge carries a confidence score and a provenance label (authoritative or inferred), enabling downstream analysis to distinguish high-confidence supply relationships grounded in production statistics from inferred relationships derived from LLM domain knowledge. We treat STDN construction as a knowledge-synthesis problem over heterogeneous technical documentation and trade data under incomplete information: manual expert curation lacks scalability, and single-query LLM prompting propagates hallucinations without mechanisms for correction or grounding. STDN-GEN addresses this with structured multi-agent debate at each extraction stage, ontology enforcement at every layer, and a hybrid data strategy that prioritizes authoritative production statistics before invoking LLM inference. Figure 2 illustrates the layer structure on a worked example.

### 4.1 STDN-GEN Extraction Pipeline

STDN-GEN constructs STDNs in three extraction stages followed by a post-processing normalization pass. Each extraction stage follows a common propose–debate–refine loop: role-specialized agents independently propose candidate edges, a deterministic debate protocol generates agreement feedback, and agents revise their proposals. The stage terminates when proposals converge under an overlap criterion or a round cap is reached. Stage 4 applies batch normalization across the full output to enforce vocabulary consistency before analysis.

#### Stage 1: Component extraction.

Given a product description for the target technology, a Component Agent proposes a set of components that decompose the technology. Proposals are grounded in specific spans of the input text and aligned with a controlled component ontology to avoid both overly generic entries (e.g., “electronics”) and overly specific ones (e.g., individual IC part numbers). Debate agents review the proposal, identify missing or miscategorized components, and generate structured feedback. The agent revises until convergence or a round cap is reached.

#### Stage 2: Materials extraction.

A Materials Agent takes the converged component set as input and proposes constituent materials for each component. Proposals are constrained to a curated materials ontology aligned with HS codes and USGS nomenclature, covering both bulk and trace materials. The same debate–refine loop applies, with debate agents reviewing from complementary perspectives: coverage, plausibility relative to domain knowledge, and consistency with the component ontology. A parallel sub-stage (Stage 2b) extracts process consumables using a lighter two-call extract-and-judge protocol, appropriate because consumables are typically less ambiguous than constituent materials; a judge agent removes hallucinated items, adjusts confidence, and caps inferred additions at confidence ≤ 0.7.

#### Stage 3: Country assignment.

Country assignments follow a USGS-first policy. Materials with coverage in the USGS Minerals Yearbook database [1] receive authoritative producer assignments at confidence ≥ 0.95, marked source=authoritative. For materials lacking USGS coverage (typically specialized industrial chemicals, alloys, or emerging materials), three Country Agents independently rank the top-10 likely producer countries. Rankings are aggregated via Borda voting (1st place = 10 pts, …, 10th place = 1 pt); total scores are summed across agents and the top-5 countries by Borda score are selected, with confidence proportional to normalized Borda strength (confidence = total_score / 30). This hybrid approach anchors the network in ground-truth production data when available and applies principled aggregation of agent judgment for gaps.

#### Stage 4: Normalization and output.

Stage 2b process-consumable outputs are recorded in the per-technology JSON exports but are not analyzed in this paper; their evaluation is deferred to future work specifically targeting manufacturing-process dependencies. After all extraction stages, each component and material name is mapped to a canonical form via the persistent vocabulary described in Section 4.4. The system emits a flat CSV (one row per *T–C–M–P* tuple, with confidence scores and provenance labels for each edge) and a nested JSON encoding the same information for direct use in analysis pipelines and visualization tools. Stage-level convergence statistics and authoritative-versus-inferred edge counts are logged as metadata.

Implementation details, including retry logic, caching, and parallelization strategy, are described in Supplementary Methods A.

### 4.2 Debate Protocol

Stages 1 and 2 use a fixed-round, agreement-driven debate protocol (Supplementary Figure 10). In Round 1, three role-specialized agents propose candidate items independently (without peer context) and assign confidence values. The system then generates deterministic agreement feedback that classifies each candidate item as consensus (proposed by all agents), majority (proposed by most), or isolated (proposed by one), and summarizes which items are well-supported versus contested. This feedback step is rule-based rather than an additional LLM call. In subsequent rounds, agents revise by selecting from the pooled, normalized candidate set and updating confidence based on peer support; restricting agents to selection-only refinement limits terminology drift across rounds. Each retained item carries grounding metadata and a confidence score adjusted for inter-agent agreement. Debate terminates when the normalized output meets the overlap criterion (Section 4.3) or the round cap *R* is reached.

Full agent prompts are provided in Supplementary Methods A.

### 4.3 Convergence Criterion

After each debate round, the system computes a Jaccard consistency score over normalized candidate names:

(1) $$\text{Consistency}\left({𝒞}_{r},{𝒞}_{r-1}\right)=\frac{\left|{𝒞}_{r}\cap {𝒞}_{r-1}\right|}{\left|{𝒞}_{r}\cup {𝒞}_{r-1}\right|}$$

where set membership is defined over canonical names (after normalization). Convergence is declared when Consistency ≥ *τ* ; otherwise debate continues up to round cap *R*. Because the candidate sets are finite and rounds are bounded, the pipeline always terminates. Analogous metrics apply for materials (matched on name and associated component) and countries (matched on name and role). The threshold *τ* controls the quality–computation trade-off: higher values require stronger agreement but may trigger more rounds. In all experiments we use *τ* = 0.95 and *R* = 3. The value *τ*=0.95 was chosen as a pragmatic default: lower thresholds (*τ* ∈ {0.85, 0.90}) admitted additional rounds that rarely changed the final canonical output in preliminary runs, while *τ*=0.99 occasionally hit the round cap without further improvement. *R*=3 bounds runtime; in practice the median run terminates within two rounds (Section 2.5). Sensitivity to these choices is an open question for future ablation.

### 4.4 Semantic Normalization and Canonical Vocabulary

Independent agents routinely surface the same concept under different names: “Li-ion Battery,” “Lithium Ion Battery,” and “Battery Pack” are all common phrasings for the same supply chain node. Without normalization, these variants reduce measured agreement and inflate component counts. STDN-GEN applies normalization after every debate round and again in Stage 4.

#### Rule-based pass.

Deterministic transformations collapse common surface variants: lowercasing, punctuation normalization, and removal of generic trailing qualifiers such as “module” or “assembly.”

#### Semantic mapping with persistent vocabulary.

Remaining unresolved variants are sent to an LLM that maps semantically equivalent names to a single canonical form. Mappings are stored in a persistent vocabulary file (canonical_vocab.json) and reused across all subsequent runs; only unseen variants incur an LLM call. Over repeated runs on related technologies, the vocabulary reduces LLM normalization calls by approximately 70% and ensures consistent naming across all extracted STDNs. Set membership for convergence checking is always evaluated over canonical names, preventing surface-form disagreements from inflating measured divergence.

The canonical vocabulary is domain-specific and accumulated incrementally: it starts empty and grows with each run, so early runs generate more LLM normalization calls while later runs benefit from the accumulated mappings. New terms from each run are merged into the shared store before the next run begins, giving the vocabulary a compounding effect over a batch. Further detail on vocabulary construction and the canonicalization procedure is provided in Supplementary Methods A.

### 4.5 LLM Judge and Validity Estimation

#### Invalid-rate counting.

We report invalid rate under two complementary counting schemes that emphasize different aspects of judge-based validity. The occurrence-based invalid rate counts every judged label across all *R* runs and all *I* agents: if a component appears in multiple runs (e.g., proposed by two of three agents in a single run and again in a subsequent run), each occurrence contributes to the denominator. Formally, for a technology *t* with judged outputs $\left\{\left(c_{ij},v_{ij}\right)\right\}$ where *c_ij_* is the *j*-th component in run *i* and *v_ij_* ∈ {0, 1} is the judge verdict (1 = invalid),

$${invalid}_{occ}(t)=\frac{\sum_{i,j}v_{ij}}{\sum_{i,j}\;1}.$$

The deduped-canonical invalid rate deduplicates components by canonical label within each (technology, configuration) before counting:

$${invalid}_{ded}(t)=\frac{\left|\{c\in canon(t)\;:\;judge(c)=1\}\right|}{\left|canon(t)\right|},$$

where canon(*t*) is the set of distinct canonical components extracted for technology *t* across all runs. Occurrence-based rates are higher because invalid components tend to recur across runs in single-agent configurations; deduped-canonical rates are lower because each invalid canonical component contributes only once. The abstract reports the occurrence-based rate (0.063 → 0.049 at *N*=3); Table 1b reports the occurrence-based median (0.011 → 0.000) as an intuitive per-technology summary of invalid-component frequency. Both views are shown side-by-side in Supplementary Figure 7; the trends agree in direction across all reported *N*.

To estimate component validity at scale without exhaustive human review, we apply an LLM judge. For each technology, the judge receives the deduped canonical component list produced by STDN-GEN across five independent runs and a structured prompt asking it to label each component as valid (a real, in-scope manufacturing component for this technology) or invalid (hallucinated, overly generic, or misclassified). Before judging, outputs across runs are deduplicated and canonicalized so that evaluation operates at the concept level rather than the surface-form level. Judgements are aggregated across runs by majority vote to yield a per-technology invalid rate.

The invalid rate is a conservative precision proxy. Components that the judge marks as invalid but that are nonetheless legitimate (false negatives of the judge) are counted against STDN-GEN, meaning the reported invalid rate overestimates the true error rate. The gold-standard validation (Section 4.6) confirms this conservative direction: judge-based recall and precision against human-annotated lists are both high, supporting the use of judge-estimated invalid rates as reliable, scalable proxies for human assessment of output quality. The full judge prompt is provided in Supplementary Methods A.

### 4.6 Gold-Standard Annotation

To validate the LLM judge, we constructed a multi-annotator gold standard for four technologies spanning microelectronics, pharmaceuticals, and biotechnology. Multiple annotators independently enumerated primary manufacturing components following written guidelines that define in-scope items (major modules and subassemblies) and exclude raw materials, consumables, overly generic terms, and subcomponents below assembly level. Individual lists were merged through consensus adjudication: disagreements were resolved by discussion until all annotators agreed on each item’s status. Merged lists were then passed through the same canonical vocabulary used by STDN-GEN so that evaluation measures concept-level rather than string-level agreement. Only Smartphone overlaps with the 60-technology microelectronics benchmark; the remaining three technologies extend validation to domains outside the benchmark. Full annotation protocol details are in Supplementary Methods A.

### 4.7 180-Technology, 3-Domain Benchmark

Quantitative experiments use a benchmark of 180 technologies spanning three manufacturing domains: 60 microelectronics technologies (semiconductor manufacturing, photonics, display, power electronics, energy storage), 60 biotechnology technologies (clinical diagnostics, genomics instrumentation, bioprocessing equipment), and 60 pharmaceuticals technologies (oral solid dosage, sterile injectable manufacturing, drug discovery instrumentation). The microelectronics list is the same set used in our earlier work [24]; the biotechnology and pharmaceutical lists were constructed by the authors using equivalent domain-expert sourcing. Each microelectronics technology was processed through the full STDN-GEN pipeline at *N* ∈ {1, 2, 3, 4, 5} with five independent runs per (technology, configuration) for the layer-wise ablation and cost–convergence sweep (Sections 2.2 and 2.5); the biotechnology and pharmaceuticals technologies were processed at *N* ∈ {1, 3} only, with five independent runs per (technology, configuration), for the cross-domain debate-effect comparison (Section 2.3). The N=2, 4, 5 configurations were not run for biotechnology or pharmaceuticals. The full technology list is provided in Supplementary Table A.

#### Statistical inference.

Confidence intervals on reported medians are computed by nonparametric bootstrap with 10,000 resamples; the resampling unit is the technology (*n*=60 for the normalization ablation; *n*=66 for the microelectronics-only debate-strength sweep, which includes three additional pharma/biotech validation technologies and three technologies present under alternative name variants; *n*=177 for the cross-domain analysis in Section 2.3, after dropping three technologies with fewer than two paired-replicate runs in either *v*1*v*1*v*1 or *d*3*v*1*v*1). CI computation uses a fixed random seed (42) for reproducibility. Paired comparisons between debate configurations (*N*=1 vs *N*=3) use the Wilcoxon signed-rank test on per-technology values (median pairwise Jaccard for stability; occurrence-based invalid rate for hallucination); we report the test statistic, two-sided *p*-value, and the count of technologies that improved, worsened, or tied. Domain-stratified analyses are reported in Section 2.3. For Table 2 (*n*=4 technologies), bootstrap CIs are reported for completeness but are too wide for reliable inference; the aggregate recall and precision figures should be read as point estimates only. All computations are implemented in scripts/stats_analysis.py and scripts/cross_domain_stability_analysis.py in the paper repository.

### 4.8 Implementation: models, sampling, and run configuration

The empirical results in Section 2 are produced by STDN-GEN running OpenAI’s hosted gpt-4.1-mini for the component, materials, and country extraction stages, and gpt-4.1 for the canonical-vocabulary mapping step and the LLM judge (Section 4.5). Component extraction uses temperature 0.03 and top-*p* 0.0001 at every *N* ∈ {1, 2, 3, 4, 5} rung; materials and country stages use top-*p* 0.0001 at the model’s default temperature (0.1 for other pipeline stages without an explicit override). The component normalization model is stronger than the extraction model because canonical-vocabulary fidelity is the dominant stability lever (Section 2.2).

The pipeline does not set the OpenAI seed parameter, so individual runs are not bit-reproducible; this is by design. Reported medians (and 95% bootstrap CIs) are computed across five independent runs per (technology, configuration), and the statistical inference described in Section 4.7 accounts for this run-level stochasticity. Per-run materials extraction performs up to ten candidate-iteration rounds, with a fuzzy-match threshold of 0.75 against the materials ontology. The country stage returns the top five producing countries by Borda-aggregated rank. Agent transient-failure retries are set to five.

The exact configurations used for the 180-technology benchmark are provided in the code repository: config_micro_d5v1v1.json for the microelectronics *N*=5 sweep (with parallel configurations for each *N*: config_micro_v1v1v1.json, config_micro_d2v1v1.json, etc.); and config_{biotech,pharma}_{v1v1v1,d3v1v1}.json for the biotechnology and pharmaceuticals *N* ∈ {1, 3} pilots. The technology lists are data/tech_list_microelectronic_products.csv, data/tech_list_biotechnology_products.csv, and data/tech_list_pharmaceutical_products.csv. The canonical-vocabulary snapshot used to produce the ablation tables (Tables 1a and 1b) is data/component_canonical_vocab_v1.0.snapshot.json; vocabulary v1.1 (with targeted refinements for the vignette technologies described in Section 2.1) is tagged in the code repository as commssustain-vocab-v1.1. All configurations, vocabulary files, and technology lists are version-controlled and publicly available at the repository URL provided in the Data Availability statement.

## Supplementary Material

1

## Figures and Tables

**Fig. 1:**
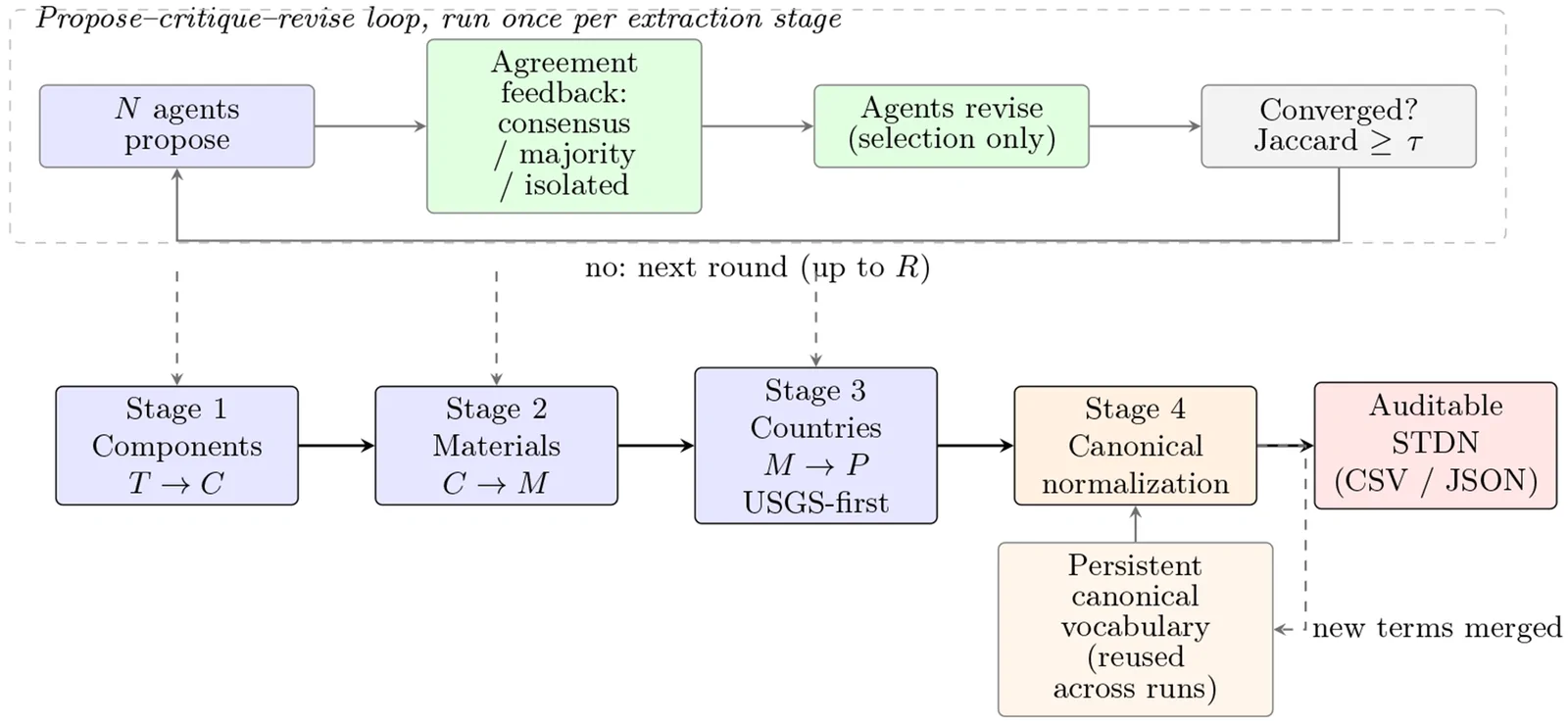
The STDN-GEN pipeline. Three extraction stages build the *T* → *C* → *M* → *P* layers in sequence; each runs the propose–critique–revise loop shown at the top, iterating until normalized agent proposals reach the convergence threshold *τ* or the round cap *R*. Stage 3 prefers authoritative USGS production statistics and falls back to Borda-aggregated agent rankings. Stage 4 maps every extracted name onto a persistent canonical vocabulary that grows across runs, yielding the auditable STDN.

**Fig. 2:**
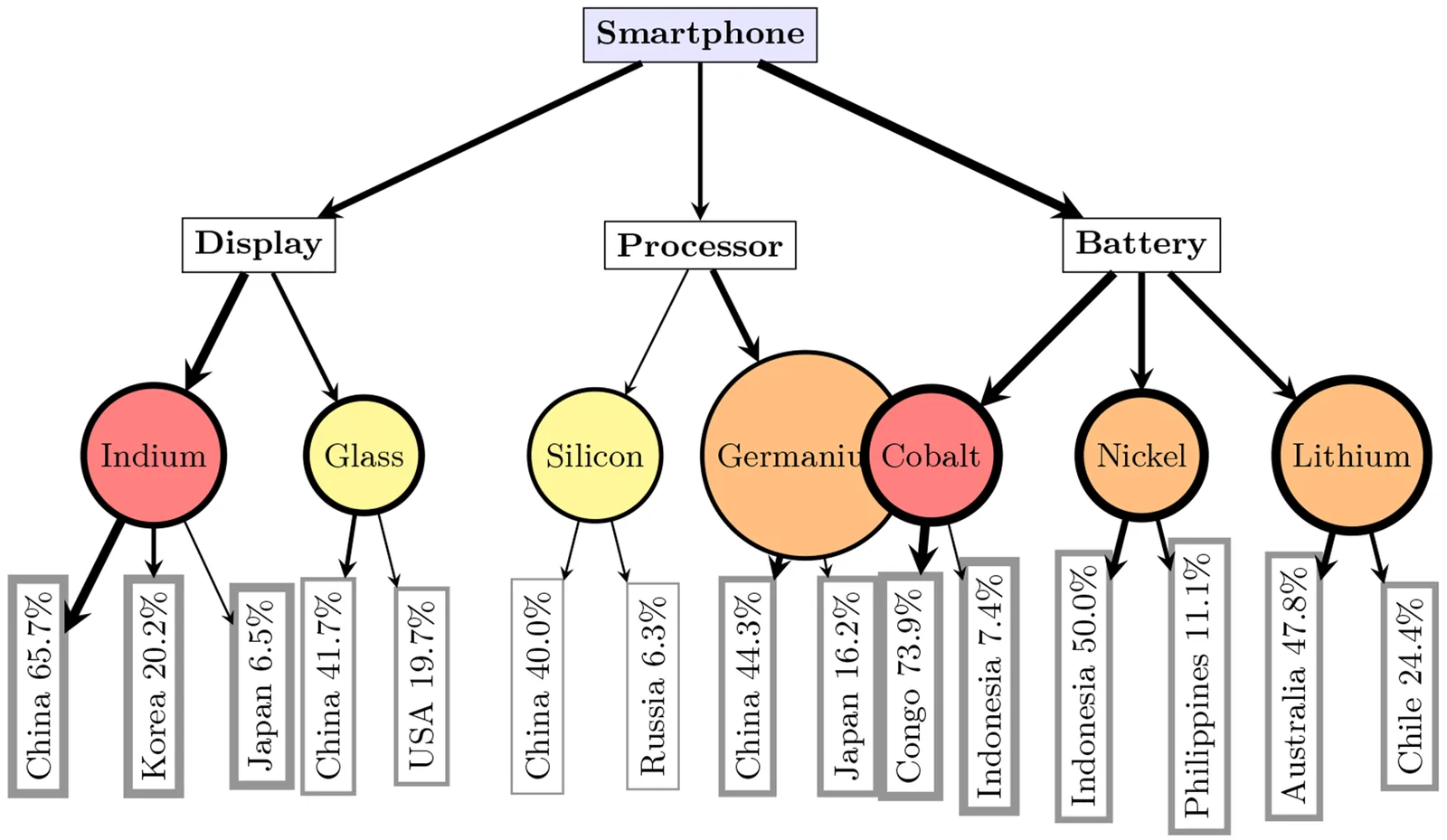
Shallow technology dependency network for smartphones. Node size scales with Herfindahl–Hirschman Index (concentration). Red nodes: extreme concentration (HHI > 5,000); orange: high (HHI 2,500–5,000); yellow: medium (HHI 2,000–2,500). Edge thickness indicates relative importance (four bins). Top producers and market share percentages are shown at the country tier. Country shares derived from STDN-GEN runs grounded in USGS Mineral Commodity Summaries 2024 (data years 2023–2024).

**Fig. 3:**
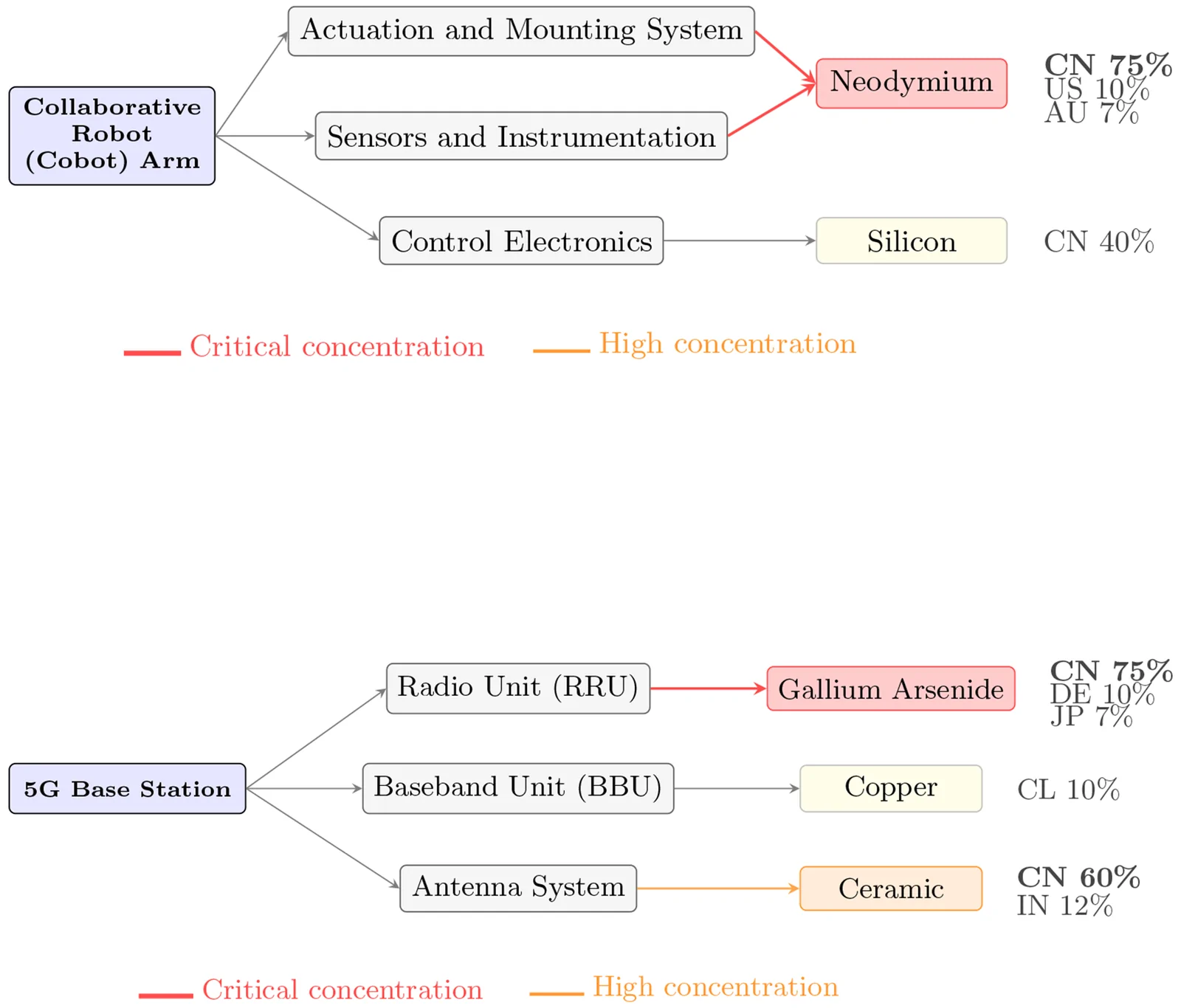
Layered STDN vignettes for a collaborative robot arm (Panel A) and a 5G base station macro cell (Panel B), illustrating that the processing-tier concentration pattern observed in the smartphone case study (Figure 2) generalises across industrial-robotics and digital-infrastructure technology classes. Each panel shows a representative subset of components, their constituent materials coloured by concentration risk (red: extreme; orange: high; unshaded: moderate), and the top producing countries with share percentages. Data from five-agent debate runs on 60 microelectronic technologies. Pipeline configurations are named by the agent count at each of the three extraction stages, so d5v1v1 denotes five debating component agents (*N*=5) followed by a single materials agent and a single country agent, and v1v1v1 denotes one agent at every stage (no debate). Country shares derived from STDN-GEN runs grounded in USGS Mineral Commodity Summaries 2024 (data years 2023–2024). (a) Collaborative robot arm. The NdFeB-magnet servo system, surfaced in the actuation and instrumentation components by five-agent debate, depends on neodymium refined predominantly in China (75% of production; United States 10%, Australia 7%). Two edges converge on the single Neodymium node because the actuation and the sensor-instrumentation subsystems depend on it independently; the shared node is what makes the material a chokepoint for the arm as a whole rather than for one subsystem. (b) 5G base station macro cell. The radio unit depends on gallium arsenide, where China accounts for 75% of production; the antenna system uses ceramics concentrated 60% in China.

**Fig. 4:**
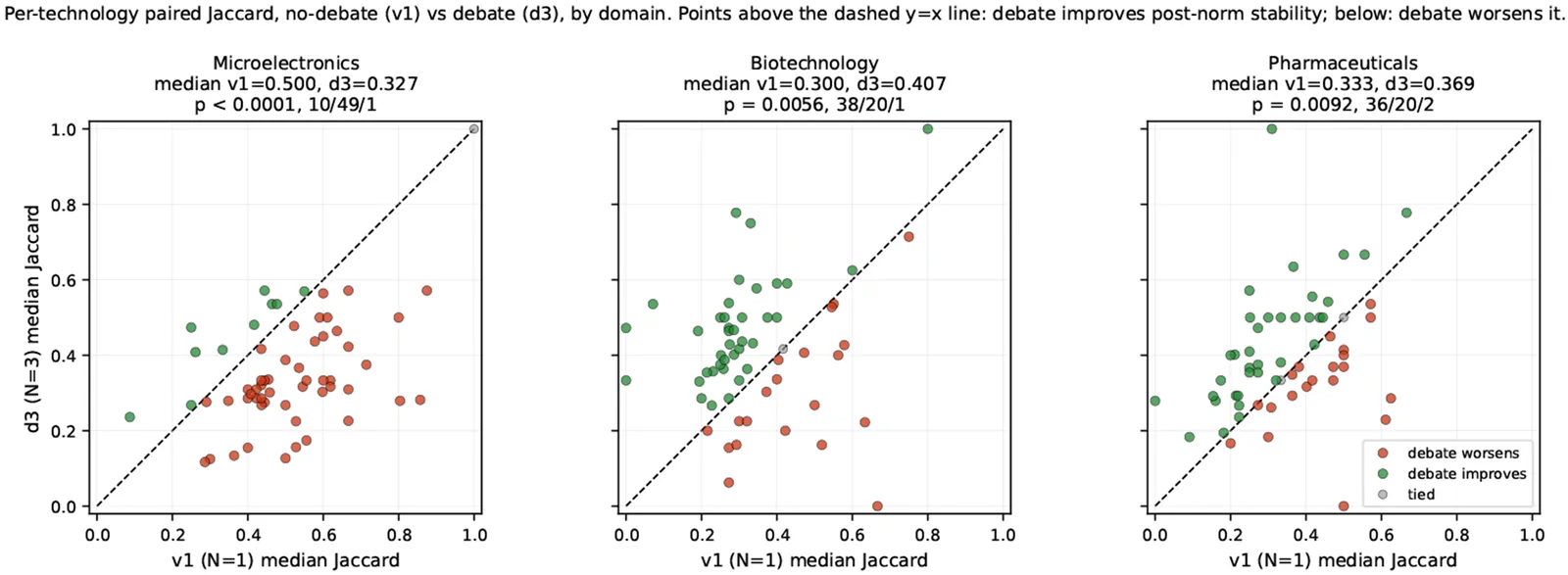
Per-technology paired post-normalization Jaccard, *v*1 (*N*=1, no debate) versus *d*3 (*N*=3, debate), by domain. Each point is one technology; the dashed line is *y* = *x*. Points above the line indicate that debate improves post-normalization stability for that technology; points below indicate debate worsens it. Microelectronics is dominated by below-line points (debate lowers post-normalization stability); biotechnology and pharmaceuticals are dominated by above-line points (debate raises post-normalization stability). Panel headers report domain medians and the paired Wilcoxon two-sided *p*-value with the improved/worsened/tied count from Table 2.

**Fig. 5:**
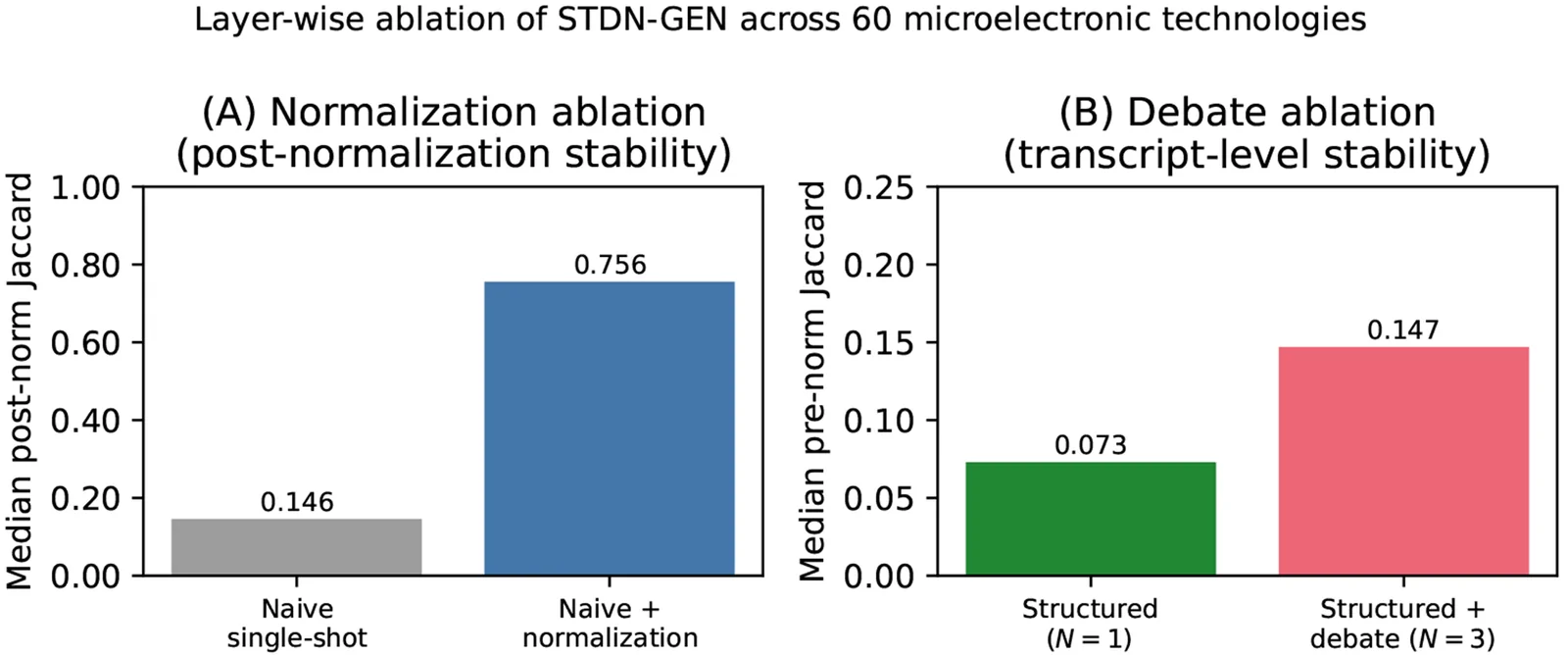
Layer-wise ablation of STDN-GEN, across 60 microelectronic technologies. (A) Normalization ablation: ontology-backed canonical mapping applied to a naive single-shot LLM output raises post-normalization Jaccard from 0.146 (95% CI 0.12–0.18) to 0.756 (95% CI 0.70–0.80) (Table 1). (B) Debate ablation in microelectronics: agreement-feedback-driven debate at *N*=3 raises transcript-level (pre-normalization) Jaccard from 0.073 (95% CI 0.055–0.089) to 0.147 (95% CI 0.123–0.166). The two panels measure complementary aspects: panel A shows normalization’s effect on output consistency; panel B shows debate’s effect on transcript-level agreement in microelectronics, the single-domain setting in which the broadening characterization holds unambiguously (see Table 2 for the cross-domain post-normalization picture).

**Fig. 6:**
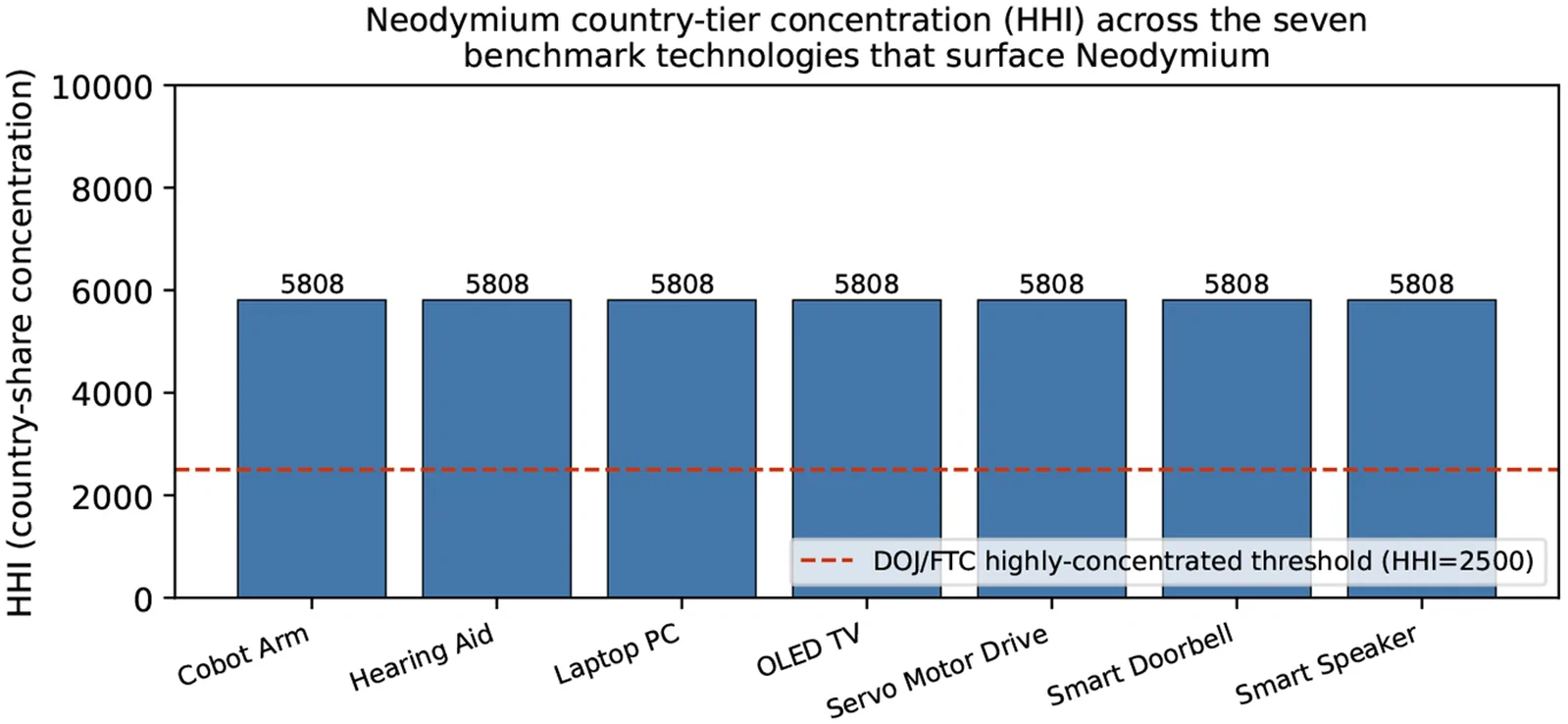
Per-technology Herfindahl–Hirschman Index (HHI) for Neodymium country-tier dependency across the seven benchmark technologies in which STDN-GEN surfaces Neodymium (union across five *N*=5 debate runs). HHI is computed as the sum of squared country production shares (0–100), so it ranges from 0 to 10,000. The dashed red line marks the US DOJ/FTC threshold for “highly concentrated” markets (HHI=2,500) widely used in critical-materials policy. All seven technologies share the same Neodymium country-share distribution (China 75%, US 10%, Australia 7%, Russia 5%, India 3%), yielding identical HHI = 5,808; the bars are plotted separately to make the technology breadth visible and to emphasize that the concentration verdict is invariant across this set of consumer, industrial, and infrastructure technologies.

**Table 1:** Normalization ablation: canonical-vocabulary mapping applied to a naive single-shot LLM output. Median pairwise Jaccard similarity is measured post-normalization (i.e., after canonical mapping), aggregated across 60 microelectronic technologies. Medians with 95% bootstrap confidence intervals over 10,000 resamples (resampling unit = technology, *n*=60). Canonical normalization alone delivers a roughly 4–5× stability improvement.

Configuration	Median Jaccard ↑	Median #components
Naive single-shot	0.146 (0.12–0.18)	39.5 (37–44)
Naive + canonical normalization	0.756 (0.70–0.80)	12.0 (11–14)

**Table 2:** Cross-domain debate ablation: paired-technology median Jaccard similarity of canonical-component sets (post-normalization) at *v*1*v*1*v*1 (no debate, *N*=1) and *d*3*v*1*v*1 (3 component agents, *N*=3) across microelectronics, biotechnology, and pharmaceuticals. Per-technology values are the median pairwise Jaccard across the available ≤ 5 runs per (technology, configuration). Δ is the domain median of (*d*3 – *v*1); “imp/wor/tied” is the per-technology count of improved, worsened, and tied technologies in the paired comparison; the Wilcoxon two-sided *p*-value is computed on per-technology (*d*3 – *v*1) differences. The combined-sample row pools paired technologies across the three domains and is included to make the cancellation visible.

Domain	*n*	*v*1 median ↑	*d*3 median ↑	Δ	imp/wor/tied	Wilcoxon *p*
Microelectronics	60	0.500	0.327	−0.173	10/49/1	< 0.0001
Biotechnology	59	0.300	0.407	+0.107	38/20/1	0.0056
Pharmaceuticals	58	0.333	0.369	+0.036	36/20/2	0.0092

Combined	177	0.400	0.369	−0.031	84/89/4	0.55

**Table 3:** Judge validation against multi-annotator gold standard on four technologies (53 unique canonical components total). Lower-bound precision is conservative because the gold standard is incomplete; manual review of apparent false positives indicates that most are legitimate components missing from the gold standard rather than judge errors; manual re-adjudication of apparent false positives suggests substantially higher precision after re-adjudication, though the validation sample is small. Technologies are Pharmaceutical Lyophilizer, Rotary Tablet Press, Single-use Bioreactor, and Smartphone, evaluated under both v1v1v1 and d3v1v1 pipeline configurations. The aggregate row reports the unweighted per-technology aggregate; bootstrap CIs are not reported for the aggregate because *n*=4 is too small for reliable bootstrap inference (see Methods).

Technology	Gold-standard #components	Judge recall ↑	Lower-bound precision ↑
Pharmaceutical Lyophilizer	9	1.000	0.360
Rotary Tablet Press	9	1.000	1.000
Single-use Bioreactor	11	1.000	1.000
Smartphone	24	0.958	0.852

Aggregate (4 technologies)	53	0.981	0.722

## Data Availability

Producer-country production statistics used for material-tier edges are drawn from the USGS Minerals Yearbook [1], which is publicly available. The 60-technology benchmark used for the layer-wise ablation and the four-technology gold-standard component lists used for judge validation are provided in Supplementary Information. All input data, intermediate outputs, derived STDN files, and the canonical-vocabulary mapping table generated during this study are publicly available in the project repository at https://github.com/dads2busy/dpi_stdn_agentic. Configurations used to produce the reported results are in the config_micro_*.json files in the code repository; the canonical-vocabulary snapshot used for the ablation tables is data/component_canonical_vocab_v1.0.snapshot.json.

## Code availability.

The STDN-GEN extraction pipeline used in this study extends the earlier STDN-GEN prototype described in [24]. Source code, configuration files, and the full pipeline implementation are publicly available at https://github.com/dads2busy/dpi_stdn_agentic. The Methods section and Supplementary Methods document the canonical-vocabulary construction, the multi-agent debate protocol, and the convergence criterion in sufficient detail for independent reimplementation. The exact configurations and the seed-less stochasticity of OpenAI hosted models are documented in Section 4.8. Authors recommend that readers cite the specific commit hash of the code repository for reproducibility; a Zenodo DOI is forthcoming.

## A Appendix A: Evaluated Technology List

Table 4 lists the 180 technologies evaluated in our cross-domain experiments, grouped by manufacturing domain and sub-domain. The microelectronics block (60 technologies across 12 sub-domains) anchors the layer-wise ablation in Section 2.2; the biotechnology and pharmaceuticals blocks (60 technologies each across 12 sub-domains) are used in the cross-domain debate-effect analysis in Section 2.3. The source CSVs are version-controlled in the code repository at data/tech_list_microelectronic_products.csv, data/tech_list_biotechnology_products.csv, and data/tech_list_pharmaceutical_products.csv.

**Table 4:** Evaluated technology set used in cross-domain STDN-GEN experiments: 180 technologies (60 microelectronics, 60 biotechnology, 60 pharmaceuticals) across three manufacturing domains.

Domain	Technology
Consumer Electronics	Smartphone
Consumer Electronics	Laptop PC
Consumer Electronics	Large-Format OLED Television
Consumer Electronics	Smart Speaker / Voice Assistant Device
Consumer Electronics	Hearing Aid

Data Center and Networking	Data Center Server
Data Center and Networking	Network Switch/Router
Data Center and Networking	5G Base Station (Macro Cell)
Data Center and Networking	Enterprise Storage Array (SAN)
Data Center and Networking	WiFi 7 Access Point

Automotive	Autonomous Vehicle Compute Platform
Automotive	Automotive ECU (Engine Control Unit)
Automotive	EV Traction Inverter
Automotive	EV On-Board Charger
Automotive	Automotive Tire Pressure Monitoring System (TPMS)

Industrial	Industrial PLC Module
Industrial	Industrial Motor Drive (Variable Frequency)
Industrial	Solar String Inverter
Industrial	Near-Infrared Spectroscopy Instrument
Industrial	Industrial Welding Robot Controller

Edge and Embedded Computing	Edge AI Inference Box
Edge and Embedded Computing	Surveillance DVR/NVR
Edge and Embedded Computing	Point-of-Sale Terminal
Edge and Embedded Computing	Digital Signage Player
Edge and Embedded Computing	Industrial IoT Gateway

Military and Defense	AESA Radar Module (Military)
Military and Defense	Thermal Imaging Sight
Military and Defense	Inertial Navigation System
Military and Defense	Military Avionics Module
Military and Defense	Electronic Warfare Jammer Pod

Space	Space-Hardened Satellite Computer
Space	Satellite Communication Terminal
Space	Star Tracker (Satellite Attitude Sensor)
Space	Space-Grade Solar Array Power Regulator
Space	CubeSat On-Board Computer

Telecommunications	Fiber-Optic Communication Terminal
Telecommunications	GPS Timing Receiver
Telecommunications	Small Cell (5G Picocell)
Telecommunications	Microwave Backhaul Radio
Telecommunications	Submarine Cable Repeater

Medical	Medical Ultrasound System
Medical	MRI Machine
Medical	CT Scanner
Medical	Pulse Oximeter
Medical	Implantable Cardiac Defibrillator (ICD)

Lighting and Photonics	LED Luminaire
Lighting and Photonics	Automotive LED Headlamp Module
Lighting and Photonics	UV-C Disinfection System
Lighting and Photonics	Laser Projector
Lighting and Photonics	Fiber-Optic Illumination System

Sensing and Security	LiDAR Sensor System
Sensing and Security	Face Recognition Access Control System
Sensing and Security	Contactless Payment Card
Sensing and Security	Automotive Night Vision System
Sensing and Security	Smart Doorbell Camera

Robotics and Motion	Collaborative Robot (Cobot) Arm
Robotics and Motion	Drone Flight Controller
Robotics and Motion	Servo Motor Drive
Robotics and Motion	CNC Machine Tool Controller
Robotics and Motion	Automated Guided Vehicle (AGV) Controller

**BIOTECHNOLOGY (60 technologies)**	
Clinical Diagnostics	Real-Time PCR Thermocycler
Clinical Diagnostics	Clinical Flow Cytometer
Clinical Diagnostics	Automated Immunoassay Analyzer
Clinical Diagnostics	Hematology Analyzer
Clinical Diagnostics	Blood Gas Analyzer

Genomics and Sequencing	Illumina Short-Read DNA Sequencer
Genomics and Sequencing	Nanopore Sequencer
Genomics and Sequencing	Microarray Scanner
Genomics and Sequencing	Capillary Electrophoresis Genetic Analyzer
Genomics and Sequencing	Single-Cell RNA-Seq Instrument

Synthetic Biology and Gene Editing	Oligonucleotide Synthesizer
Synthetic Biology and Gene Editing	Benchtop DNA Printer
Synthetic Biology and Gene Editing	CRISPR Electroporation System
Synthetic Biology and Gene Editing	Automated Molecular Cloning Workstation
Synthetic Biology and Gene Editing	Gene Assembly Platform

Cell and Gene Therapy Manufacturing	Closed-System Cell Expansion Bioreactor
Cell and Gene Therapy Manufacturing	Viral Vector Production Bioreactor
Cell and Gene Therapy Manufacturing	Cell Washing and Concentration System
Cell and Gene Therapy Manufacturing	Cryopreservation Controlled-Rate Freezer
Cell and Gene Therapy Manufacturing	Lentiviral Transfection System

Biopharmaceutical Production	Single-Use Stirred-Tank Bioreactor (2000L)
Biopharmaceutical Production	Continuous mRNA Manufacturing System
Biopharmaceutical Production	Stainless Steel Fed-Batch Bioreactor
Biopharmaceutical Production	Vaccine Fill-Finish Line
Biopharmaceutical Production	Monoclonal Antibody Perfusion Bioreactor

Drug Delivery Systems	Lipid Nanoparticle Encapsulation System
Drug Delivery Systems	Prefilled Syringe Autoinjector
Drug Delivery Systems	Implantable Drug Infusion Pump
Drug Delivery Systems	Metered-Dose Inhaler
Drug Delivery Systems	Transdermal Patch Manufacturing System

Bioprocessing Equipment	Tangential Flow Filtration Cassette System
Bioprocessing Equipment	Protein A Chromatography Column
Bioprocessing Equipment	Industrial Freeze Dryer (Lyophilizer)
Bioprocessing Equipment	Continuous Centrifuge Separator
Bioprocessing Equipment	Sterile Filtration Skid

Agricultural Biotechnology	Plant Transformation Gene Gun
Agricultural Biotechnology	Seed Trait Analysis Platform
Agricultural Biotechnology	Biopesticide Fermentation Unit
Agricultural Biotechnology	Tissue Culture Growth Chamber
Agricultural Biotechnology	Phenotyping Imaging Robot

Industrial Fermentation	Industrial Enzyme Fermenter (50000L)
Industrial Fermentation	Bioethanol Distillation Column
Industrial Fermentation	PHA Bioplastic Extractor
Industrial Fermentation	Yeast Propagation Tank
Industrial Fermentation	Algae Photobioreactor

Point-of-Care and Biosensors	Continuous Glucose Monitor
Point-of-Care and Biosensors	Lateral Flow Immunoassay Reader
Point-of-Care and Biosensors	Portable Blood Coagulation Meter
Point-of-Care and Biosensors	Handheld Pulse Oximeter
Point-of-Care and Biosensors	Rapid Molecular Diagnostic Cartridge System

Biomedical Imaging	Confocal Laser Scanning Microscope
Biomedical Imaging	Cryo-Electron Microscope
Biomedical Imaging	Small Animal PET/CT Scanner
Biomedical Imaging	Super-Resolution Fluorescence Microscope
Biomedical Imaging	Digital Pathology Slide Scanner

Laboratory Automation	Automated Liquid Handling Robot
Laboratory Automation	High-Throughput Microplate Reader
Laboratory Automation	Robotic Colony Picker
Laboratory Automation	Automated Nucleic Acid Extraction System
Laboratory Automation	Laboratory Sample Storage Freezer (−80C)

**PHARMACEUTICALS (60 technologies)**	
Oral Solid Dosage	Rotary Tablet Press
Oral Solid Dosage	Hard Capsule Filling Machine
Oral Solid Dosage	Film Coating System
Oral Solid Dosage	Dissolution Testing Apparatus
Oral Solid Dosage	Tablet Deduster and Metal Detector

Sterile Injectable Manufacturing	Aseptic Vial Filling Line
Sterile Injectable Manufacturing	Ampoule Forming and Sealing Machine
Sterile Injectable Manufacturing	IV Bag Manufacturing System
Sterile Injectable Manufacturing	Autoclave Sterilizer (Pharmaceutical Grade)
Sterile Injectable Manufacturing	Isolator-Based Sterility Test System

API Synthesis	Glass-Lined Chemical Reactor
API Synthesis	High-Pressure Hydrogenation Vessel
API Synthesis	Rotary Vacuum Dryer
API Synthesis	Centrifugal Solids Discharge Filter
API Synthesis	Wiped Film Evaporator

Pharmaceutical Packaging	Blister Packaging Machine
Pharmaceutical Packaging	Bottle Filling and Capping Line
Pharmaceutical Packaging	Serialization and Track-and-Trace System
Pharmaceutical Packaging	Cartoning Machine
Pharmaceutical Packaging	Tamper-Evident Seal Applicator

Continuous Manufacturing	Continuous Flow Reactor System
Continuous Manufacturing	Twin-Screw Wet Granulator (Continuous)
Continuous Manufacturing	Continuous Direct Compression Press
Continuous Manufacturing	In-Line PAT (Process Analytical Technology) Probe
Continuous Manufacturing	Continuous Crystallization Unit

Topical and Transdermal	Vacuum Emulsifying Mixer (Cream/Ointment)
Topical and Transdermal	Transdermal Patch Laminator
Topical and Transdermal	Colloid Mill (Topical Formulation)
Topical and Transdermal	Ointment Filling and Sealing Machine
Topical and Transdermal	Dermal Penetration Test Apparatus (Franz Cell)

Controlled Release Formulation	Fluid Bed Pellet Coater
Controlled Release Formulation	Osmotic Tablet Core Press
Controlled Release Formulation	Microsphere Encapsulation System
Controlled Release Formulation	Hot-Melt Extrusion System
Controlled Release Formulation	Wurster Column Coating Insert

Pharmaceutical Quality Control	HPLC System (Pharmaceutical Grade)
Pharmaceutical Quality Control	Karl Fischer Titrator
Pharmaceutical Quality Control	Particle Size Analyzer (Laser Diffraction)
Pharmaceutical Quality Control	Friability Tester
Pharmaceutical Quality Control	Endotoxin Detection System (LAL/rFC)

Pharmaceutical Water and Clean Utilities	Water for Injection (WFI) Distillation Unit
Pharmaceutical Water and Clean Utilities	Reverse Osmosis/Electrodeionization Purification System
Pharmaceutical Water and Clean Utilities	Clean Steam Generator
Pharmaceutical Water and Clean Utilities	TOC (Total Organic Carbon) Analyzer
Pharmaceutical Water and Clean Utilities	Sanitizable Distribution Loop Skid

Powder Processing and Granulation	Fluid Bed Dryer/Granulator
Powder Processing and Granulation	Cone Mill (Screening/Sizing)
Powder Processing and Granulation	V-Blender (Powder Mixing)
Powder Processing and Granulation	Jet Mill (Micronization)
Powder Processing and Granulation	Roller Compactor (Dry Granulation)

Clinical Trial Supply	Small-Scale Encapsulator (Clinical Batches)
Clinical Trial Supply	Clinical Labeling and Randomization System
Clinical Trial Supply	Temperature-Controlled Shipping Container
Clinical Trial Supply	Interactive Response Technology (IRT) Dispensing Unit
Clinical Trial Supply	Comparator Sourcing and Over-Encapsulation Machine

Radiopharmaceutical Production	Medical Cyclotron
Radiopharmaceutical Production	Hot Cell Shielded Workstation
Radiopharmaceutical Production	PET Tracer Automated Synthesis Module
Radiopharmaceutical Production	Radiochemical Dose Calibrator
Radiopharmaceutical Production	Technetium-99m Generator

### Appendix Figures (Optional)

If space permits, we include supplemental figures that provide additional context on validity estimates under different counting schemes, technology-level variability, and robustness heterogeneity.
