# Supplementary material for: STDN-GEN: rapid synthesis of layered critical-material dependency networks for supply-chain sustainability analysis
Source: Res Sq. 2026 Sep 10:rs.3.rs-10767641. Preprint. [Version 1] doi: 10.21203/rs.3.rs-10767641/v1 (PMC13622788; doi:10.21203/rs.3.rs-10767641/v1)
Supplement: Supplementary file 1 [file NIHPPrs10767641v1-supplement-1.pdf]

Supplementary Material: Methodological Details

This supplementary section records methodological details referenced in Section 4. It includes (i) agent roles and specifications, (ii) semantic normalization and canonicalization procedures, (iii) debate convergence metrics and termination arguments, and (iv) fallback country inference and rank aggregation. The intent is to preserve reproducibility and implementability while keeping the main paper focused on the architectural decisions and debate protocol.

S.0 Implementation details: retry logic, caching, and parallelization

Full implementation details (retry configuration, caching layers, and parallelization strategy) are available in the project repository at https://github.com/dads2busy/dpi stdn agentic; this section provides a brief summary. STDN-GEN uses configurable per-agent retry logic (default five retries with exponential back-off) to handle transient LLM API failures. Two caching layers reduce redundant LLM calls: a short-lived material cache (default TTL 24 h) and a persistent LLM fallback cache for country-inference results (default TTL 720 h). Parallelization is managed at the technology level: a configurable concurrency limit (default 10) controls how many technologies are processed simultaneously within a single run; a separate parallel-runs script spawns independent OS-level subprocesses for ensemble experiments.

S.1 Agent roles, interfaces, and prompting requirements

STDN-GEN instantiates multiple LLM-based agents. All agents share a common input/output contract and differ primarily by role conditioning (system prompt),

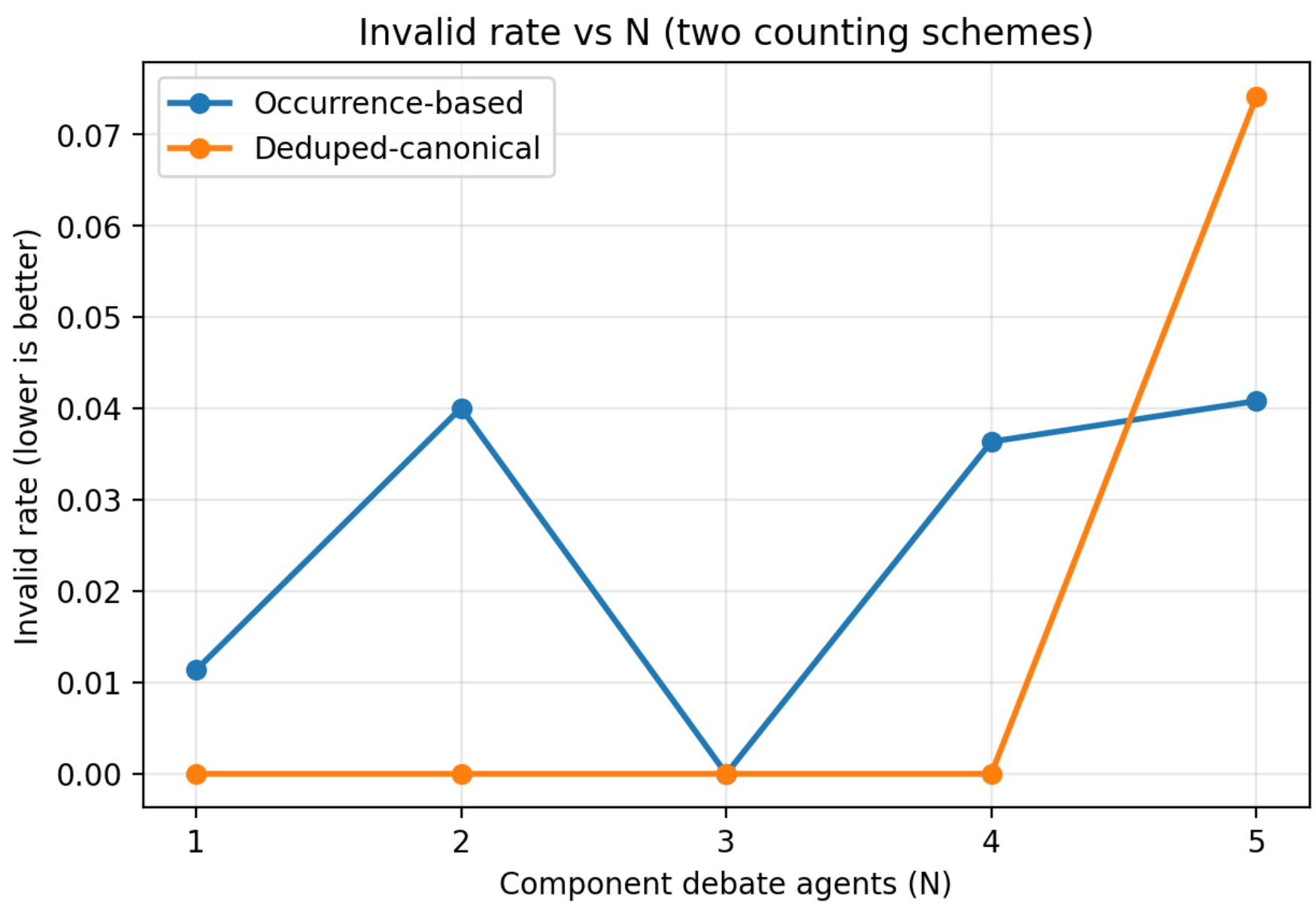

Fig. 7: **Invalid rate under two counting schemes.** Occurrence-based invalid rate counts all judged outputs (including repeats across runs), while deduped-canonical invalid rate deduplicates by canonical component within each (technology, configuration). Trends consistent across both views strengthen the interpretation that debate reduces hallucination-like errors (components judged not plausible by an independent LLM judge). The abstract reports the occurrence-based rate (0.063 → 0.049 at N=3); Table 1b reports the deduped-canonical rate (0.011 → 0.000); see Methods for definitions.

permitted tools (e.g., ontology lookup or external data queries), and stage-specific constraints.

Common input context.

Agents receive:

- Target technology identifier and a natural-language technology description.
- Stage-specific context:
  - Stage 1: none (technology-only).
  - Stage 2: stabilized component list from Stage 1.
  - Stage 3: stabilized materials list from Stage 2.
- Canonical vocabularies (if available): component and/or material canonical name lists and previously learned mappings.
- Optional structured evidence (if available): ontology entries, USGS production statistics, and cached prior results.

Common output schema.

For Stages 1–2, each agent returns a set of items, each with:

- **Name:** a short noun phrase for the component/material.
- **Optional grounding:** a short justification or text span pointer when the input description supports it.
- **Confidence:** a real-valued score in $[0, 1]$ representing the agent's belief that the item is a valid dependency at the intended granularity.

For Stage 3 fallback inference, each agent returns a ranked list of candidate producer countries for a material, typically top-K with $K = 10$.

S.1.1 Extraction agents

Component Extraction Agent (Stage 1).

Objective: propose primary manufacturing components at an appropriate level of abstraction, avoiding both overly generic entries (e.g., "electronics") and overly specific sub-parts (e.g., single resistor types) unless the technology warrants it.

Constraints and expectations:

- Prefer manufacturing-relevant components (assemblies or major modules).
- When an ontology is available, select the closest ontology-aligned label; otherwise propose a best-effort descriptive label.
- Avoid duplicates and near-duplicates (handled by normalization, but discouraged at the source).

Materials Extraction Agent (Stage 2).

Objective: propose materials required to manufacture each component. The agent should cover both bulk materials (e.g., aluminum) and critical trace materials where relevant (e.g., indium in ITO).

Constraints and expectations:

- Materials should be plausibly used in manufacturing the component class (not necessarily in every design variant).
- Prefer ontology-aligned materials where possible (e.g., HS-aligned materials list).
- Where the input text is underspecified, the agent may rely on general domain knowledge but should reflect this uncertainty in confidence.

Country Profiling Agent (Stage 3 fallback mode).

Objective: when authoritative coverage is not available, independently propose a ranked list of likely producing countries for a material.

Constraints and expectations:

- Return a ranked list of distinct countries (top-K, typically $K = 10$).

- Rankings should reflect production/refining capability for the material (as appropriate to the material definition used in the network).
- Use conservative confidence; the system will aggregate multiple lists.

Process Consumables Extraction Agent (Stage 2b).

Objective: propose materials consumed during manufacturing that do not become part of the final product (e.g., process gases, etchants, solvents, photoresists, CMP slurries, cooling media).

Constraints and expectations:

- Materials must be plausibly consumed in the manufacturing process for the technology class.
- The agent receives the stabilized component list as context for inferring manufacturing processes, but does not produce component-level associations.
- The materials ontology is used as a reference, not a hard constraint—many process consumables (photoresists, specialty gases) may not appear in the HS-aligned ontology.
- Constituent materials (those that physically become part of the product) must not be included.

Process Consumables Judge (Stage 2b).

Objective: review the extraction agent's proposal, removing misclassified or hallucinated items, adjusting confidence, and adding missing process consumables.

Constraints and expectations:

- The judge may remove, adjust, or keep any proposed item, and may add new items.
- Additions are capped at confidence ≤ 0.7 unless strongly justified.
- Each action (keep, remove, adjust, add) must include a brief justification.

Stage 2b uses no debate loop—it is a single-pass protocol (one extraction call, one judge call). Final confidence is the judge's output confidence directly, with no peer-support multiplier.

S.1.2 Debate agents

For Stages 1 and 2, the debate uses $N = 3$ agents with different role prompts to encourage diverse critiques. A representative configuration is:

- **Supply-chain perspective:** emphasizes procurement realism, supplier structure, and manufacturing plausibility.
- **Manufacturing/assembly perspective:** emphasizes physical integration and what must exist as a manufacturing module.

- **Classification/coverage perspective:** emphasizes taxonomy consistency, coverage completeness, and naming clarity.

Round 1 proposals are independent (agents do not see peers). In later rounds, agents see the candidate pool and system-generated agreement feedback (Section 4.2), but are restricted to selection-only refinement (Section A).

S.1.3 Confidence scale and guidance

Agents are instructed to produce confidence values in $[0, 1]$ and to use consistent semantics:

- Higher confidence for near-universal components/materials for a technology class or when supported by authoritative sources.
- Lower confidence when presence is design-dependent, geographically contingent, or weakly supported by the provided text.

Implementations may use a discretized banding (e.g., 7-level bins) for interpretability, but the output is stored as a continuous scalar to support aggregation and thresholding downstream.

S.2 Debate protocol details and selection-only refinement

S.2.1 Deterministic agreement feedback

Given N agent outputs in a round, the system computes an agreement summary for each unique canonicalized item:

- n_{support}: number of agents that selected/proposed the item.
- $\bar{s}$: mean confidence over supporting agents.

Support labels are assigned by thresholding n_{support}:

- **Consensus:** $n_{\text{support}} = N$.
- **Majority:** $n_{\text{support}} = N - 1$ (for $N = 3$, this is 2).
- **Isolated:** $n_{\text{support}} = 1$.

The feedback text is generated by fixed templates (not by an LLM) to ensure auditability and to reduce variability across runs.

S.2.2 Selection-only refinement

In rounds $r \geq 2$, each agent must output a subset of the candidate pool formed from the prior round's union:

$$\mathcal{S}^{(r-1)} = \bigcup_{i=1}^{N} P_i^{(r-1)}.$$

After canonicalization (Section A), agents select from $\mathcal{S}_{\text{canon}}^{(r-1)}$ rather than generating new surface forms. This constraint is used to:

- reduce terminology drift (paraphrase-induced disagreement);
- bound the effective search space for convergence; and
- make overlap metrics meaningful across rounds.

S.2.3 Confidence aggregation with peer support

Final item confidence is computed by combining an agent-provided confidence with a peer-support factor. A representative form (used in our implementation for Stages 1–2) is:

$$s_{\text{final}} = s_{\text{agent}} \times \left(0.7 + 0.3 \cdot \frac{n_{\text{support}}}{N}\right),$$

where n_{support} is the support count in the final round. This preserves the agent's internal uncertainty while up-weighting consensus items and down-weighting isolated items.

S.3 Semantic normalization and canonicalization

Multi-agent extraction produces surface-level naming variation (e.g., "Li-ion battery" vs. "Lithium-ion Battery"). STDN-GEN canonicalizes names between rounds and in post-processing to (i) avoid spurious disagreement during convergence checks and (ii) support cross-technology comparisons.

S.3.1 Rule-based normalization

Before semantic mapping, the system applies deterministic string normalization:

- lowercasing;
- replacing hyphens/underscores with spaces;
- stripping common suffixes (e.g., "module", "unit", "assembly") when they do not change meaning; and
- trimming whitespace and collapsing multiple spaces.

S.3.2 LLM semantic mapping

When rule-based normalization is insufficient, the system performs semantic mapping by querying an LLM with a structured prompt that:

- groups semantically equivalent variants;
- selects a canonical form (preferring clear, specific labels); and
- returns a mapping from normalized variants to canonical names.

S.3.3 Persistent vocabulary and caching

Mappings are stored in a persistent vocabulary file (e.g., `canonical_vocab.json`). On subsequent runs, the system:

1. applies rule-based normalization;
2. checks whether the normalized form exists in the persistent mapping; and
3. only queries the LLM for unmapped variants.

This amortizes cost and improves consistency across technologies and repeated runs.

S.4 Convergence criteria and termination

S.4.1 Overlap metric

Debate convergence is evaluated by overlap between consecutive round outputs, computed over canonicalized names. For a stage output set $\mathcal{X}_r$ at round r, we use a Jaccard-style similarity:

$$\mathrm{Overlap}(\mathcal{X}_r, \mathcal{X}_{r-1}) = \frac{|\mathcal{X}_r \cap \mathcal{X}_{r-1}|}{|\mathcal{X}_r \cup \mathcal{X}_{r-1}|}.$$

Analogous metrics can be defined for structured tuples, e.g., (component, material) pairs.

S.4.2 Stopping rule

The debate loop stops if either:

- $\mathrm{Overlap}(\mathcal{X}_r, \mathcal{X}_{r-1}) \geq \tau$ for a threshold τ (e.g., $\tau = 0.95$), or
- the maximum number of rounds R is reached (e.g., $R = 3$).

S.4.3 Termination argument

Termination is guaranteed operationally by the explicit round cap R. Additionally, selection-only refinement implies that after Round 1 the candidate universe is fixed to a finite set $\mathcal{S}^{(1)}_{\text{canon}}$, so all later-round proposals are subsets of a finite set. This supports predictable behavior and bounded compute.

S.5 Stage 3 fallback: country inference and rank aggregation

When authoritative coverage is unavailable for a material, STDN-GEN infers producer countries using independent ranked lists and rank aggregation.

Independent ranked lists.

Three agents (or three independent runs of a role-conditioned agent) each output a ranked list of K countries for a material (typically $K = 10$), without observing peer outputs.

Borda aggregation.

For each agent list, the country at rank j receives $K - j + 1$ points (rank 1 receives K points). Points are summed across agents:

$$\text{score}(c) = \sum_{i=1}^{N} \text{points}_i(c).$$

Countries are then sorted by score(c) and the top-k are selected (typically $k = 5$).

Confidence for aggregated outputs.

A simple normalization is:

$$s(c) = \frac{\text{score}(c)}{N \cdot K},$$

which maps to $[0, 1]$ and increases with agreement and rank strength. These edges are labeled as `source=inferred` (or equivalent) to preserve provenance.

S.6 Output artifacts and metadata

Each run emits:

- A CSV with one row per (technology, component, material, producer) tuple, including confidence and provenance.
- A JSON encoding of the same STDN structure for downstream tooling.
- Stage-level metadata: number of rounds, final overlap, and counts of authoritative vs. inferred edges.

These artifacts support re-analysis, ablation comparisons, and audit of how an STDN was produced under a given configuration.

S.7 Debate-flow diagram

Figure 10 visualizes the per-round flow of the agreement-driven debate protocol described in Section 4.2. The diagram traces the propose–debate–refine loop: three role-specialized agents propose candidate items independently in Round 0; if the candidate sets satisfy the convergence criterion (Section 4.3), the debate ends; otherwise the system generates rule-based agreement feedback and the agents revise.

S.8 LLM judge prompt

The full judge prompt used for component validity estimation (Section 4.5) is available in the project repository at https://github.com/dads2busy/dpi_stdn_agentic. Briefly, the judge receives a technology name, the deduplicated canonical component list extracted by STDN-GEN, and structured instructions to classify each component as valid (a real, in-scope primary manufacturing component for that technology) or invalid (hallucinated, overly generic, or misclassified). The judge is instructed to use

conservative labeling so that borderline cases are counted as valid, preserving the conservative-precision property described in Section 4.5.

S.9 Gold-standard annotation protocol

The gold-standard annotation protocol used for judge validation (Section 4.6) is described in full in the project repository at https://github.com/dads2busy/dpi_stdn_agentic. Multiple annotators independently enumerated primary manufacturing components for each of the four target technologies following written guidelines that define in-scope items (major modules and subassemblies) and exclude raw materials, consumables, overly generic terms, and sub-assembly components. Individual annotator lists were merged through consensus adjudication; disagreements were resolved by discussion until all annotators agreed on each item's inclusion status. Merged lists were passed through the STDN-GEN canonical vocabulary before comparison to ensure concept-level rather than string-level evaluation.

S.10 Detailed cost and convergence plots

S.11 Occurrence-based vs deduped-canonical invalid rate

The two invalid-rate counting schemes used in the main text are compared in Figure 7 (Appendix Figures). The directions agree across all configurations; the absolute values differ because the deduped-canonical view collapses repeated invalid components within a technology to a single contribution.

S.12 CRM list coverage by the 60-technology microelectronics benchmark

Table 5 reports the fraction of materials on the USGS 2022 critical-minerals list [1] and the EU 2024 Critical Raw Materials list [5] that are surfaced by the 60-technology microelectronics benchmark, computed as the union of the five repeated N=5 STDN-GEN runs. A material is counted as surfaced if the extracted material name contains the CRM name as a substring; rare-earth and platinum-group entries listed as families in the EU regulation are decomposed into their constituent elements before matching (e.g., Heavy Rare Earth Elements becomes the element-level entries Dy, Er, Eu, Gd, Ho, Lu, Sm, Tb, Tm, Yb, Y).

Overall, 25 of 50 USGS-listed minerals (50%) and 24 of 51 EU-listed minerals (47%) are surfaced by the benchmark. The materials that are not surfaced fall into three groups. First, rare-earth and platinum-group element families that the model is not consistently naming at the element level (e.g., Cerium, Lanthanum, Praseodymium, Yttrium, Palladium, Rhodium, Ruthenium); these appear under aggregate labels such as "rare earth elements" or "catalysts" rather than as elemental entries, and are recovered by post-hoc expansion in the canonical vocabulary. Second, materials whose dominant downstream uses sit outside the microelectronics benchmark (e.g., Coking Coal, Phosphate Rock, Bauxite, Feldspar, Strontium, Fluorspar, Chromium, Hafnium, Barite/Baryte); these would be expected to surface in photovoltaic, wind, electric-vehicle battery, refractory, and chemicals benchmarks

not in this microelectronics-focused set. Third, low-volume specialty materials with few microelectronics-domain uses surveyed in the benchmark (e.g., Bismuth, Antimony, Tellurium, Samarium, Scandium); these would surface in extended benchmarks that include thermoelectrics, soldering alloys, and aerospace alloys. The pipeline architecture does not depend on the tech list; broadening the benchmark to the missing technology classes is a configuration change rather than a pipeline change.

Cross-domain coverage (suggestive).

The biotechnology and pharmaceuticals tech lists used for the cross-domain debate-effect analysis (Section 2.3) surface materials typical of bioprocess and dosage manufacturing that the microelectronics-only benchmark does not flag. Pharma-side coverage adds Helium (cryogenics in lyophilization and analytical instrumentation), Fluorspar (fluoropolymer tubing and gaskets), and Phosphorus (excipient and pH-buffering chemistry); biotech-side coverage adds bulk specialty steels (300-series stainless for hygienic vessels and piping), biocompatible polymers (PTFE, silicone, single-use bioreactor films), and pharmaceutical-grade glass (Type I borosilicate for vial filling lines). A full re-coverage analysis at the granularity of Table 5 is deferred to a follow-up; the cross-domain runs were configured to test the debate-effect direction rather than to refresh the CRM coverage audit, and the canonical vocabulary already grew during the pilot in ways that would require a fresh substring-match pass to be reported precisely.

Table 5: CRM list coverage by the 60-technology microelectronics benchmark. "USGS" = on the USGS 2022 critical-minerals list. "EU CRM" = on the EU Critical Raw Materials Act 2024 list. "Surfaced" = at least one occurrence in the union of five N=5 STDN-GEN runs across the 60-technology microelectronics benchmark. "# techs" = number of distinct benchmark technologies in which the material is surfaced.

Material	USGS	EU CRM	Surfaced	# techs
Aluminum	✓	–	✓	60
Antimony	✓	✓	–	0
Arsenic	✓	✓	✓	1
Barite	✓	–	–	0
Baryte	–	✓	–	0
Bauxite	–	✓	–	0
Beryllium	✓	✓	✓	1
Bismuth	✓	✓	–	0
Boron	–	✓	✓	4
Cerium	✓	✓	–	0
Cesium	✓	–	✓	1
Chromium	✓	–	–	0
Cobalt	✓	✓	✓	5
Coking Coal	–	✓	–	0
Copper	–	✓	✓	60
Dysprosium	✓	✓	✓	1

continued on next page

Table 5 continued from previous page

**Material**	**USGS**	**EU CRM**	**Surfaced**	**# techs**
Erbium	✓	✓	–	0
Europium	✓	✓	–	0
Feldspar	–	✓	–	0
Fluorspar	✓	✓	–	0
Gadolinium	✓	✓	–	0
Gallium	✓	✓	✓	32
Germanium	✓	✓	✓	6
Graphite	✓	–	✓	10
Hafnium	✓	✓	–	0
Helium	–	✓	✓	1
Holmium	✓	✓	–	0
Indium	✓	–	✓	17
Iridium	✓	✓	✓	1
Lanthanum	✓	✓	–	0
Lithium	✓	✓	✓	11
Lutetium	✓	✓	–	0
Magnesium	✓	✓	✓	2
Manganese	✓	✓	✓	1
Natural Graphite	–	✓	✓	10
Neodymium	✓	✓	✓	6
Nickel	✓	✓	✓	17
Niobium	✓	✓	✓	1
Palladium	✓	✓	–	0
Phosphate Rock	–	✓	–	0
Phosphorus	–	✓	✓	7
Platinum	✓	✓	✓	2
Praseodymium	✓	✓	–	0
Rhodium	✓	✓	–	0
Rubidium	✓	–	–	0
Ruthenium	✓	✓	–	0
Samarium	✓	✓	–	0
Scandium	✓	✓	–	0
Silicon metal	–	✓	✓	60
Strontium	–	✓	–	0
Tantalum	✓	✓	✓	9
Tellurium	✓	–	–	0
Terbium	✓	✓	–	0
Thulium	✓	✓	–	0
Tin	✓	–	✓	30
Titanium	✓	–	✓	19
Titanium metal	–	✓	✓	17
Tungsten	✓	✓	✓	6
Vanadium	✓	✓	✓	2

continued on next page

Table 5 continued from previous page

Material	USGS	EU CRM	Surfaced	# techs
Ytterbium	✓	✓	–	0
Yttrium	✓	✓	–	0
Zinc	✓	–	✓	2
Zirconium	✓	–	✓	1

S.13 Cross-domain stability analysis

This subsection records the methodology for the cross-domain debate-effect analysis reported in Section 2.3 (Table 2, Figure 4). The analysis script is `scripts/cross_domain_stability_analysis.py` in the paper repository, and the regeneration script for Figure 4 is `figures/fig6_cross_domain_debate_make.py`; both consume the normalized output CSVs in the code repository at `output/normalized/stdns_output_{micro,biotech,pharma}_{v1v1v1,d3v1v1}_*.csv`.

Metric.

For each (technology, configuration) combination, we compute the median pairwise Jaccard similarity of canonical-component sets across the available runs (post-normalization). With $k \leq 5$ runs per (technology, configuration), there are $\binom{k}{2}$ run-pair Jaccards per (technology, configuration); the median collapses these into one scalar per (technology, configuration). Technologies with fewer than two paired-replicate runs in either configuration are dropped from the paired comparison; this affects three technologies (one biotechnology, two pharmaceuticals), yielding 177 paired technologies in the combined sample (60 microelectronics, 59 biotechnology, 58 pharmaceuticals).

Paired test.

Within each domain we run a paired Wilcoxon signed-rank test (two-sided, `zero_method='wilcox'`) on the per-technology $(d3 - v1)$ differences. We report the test statistic W, the two-sided p-value, and the count of technologies that improved $(d3 > v1)$, worsened $(d3 < v1)$, or tied at numerical resolution. The combined-sample test pools per-technology differences across the three domains; we report it not as the headline finding but to make visible that the per-domain effects cancel when aggregated.

Bootstrap CI.

We compute a 95% bootstrap confidence interval for the combined median$(d3 - v1)$ using 10,000 resamples with a fixed seed (`numpy.random.seed(42)`). The reported interval is $(-0.0359, +0.0333)$, straddling zero, consistent with the combined-sample Wilcoxon test failing to reject.

Caveats.

The biotechnology and pharmaceuticals pilots use a single non-trivial debate strength (N=3); whether the helping-direction effect grows or saturates with larger N is not characterized here. Some pharmaceuticals technologies completed fewer than five $v1$

replicates (scheduling artefact); per-technology medians are computed over whatever replicates each technology has. This adds noise to per-tech estimates but does not bias the direction of the paired-difference test, because the same technology contributes a single matched pair of values to the test regardless of replicate count. The post-norm metric is the only stability metric reported on the expanded 180-technology benchmark; transcript-level (pre-normalization) Jaccard on biotechnology and pharmaceuticals is left to future work.

S.14 Vocabulary maturity by domain and frozen-vocabulary sensitivity

The canonical vocabulary that drives normalization grew incrementally during the cross-domain pilot. Microelectronics was normalized first (2026-04-14 to 2026-04-15) against a pre-pilot vocabulary of 2,834 entries (snapshot `component_canonical_vocab_global_primary.backup_pre_goldstandard.json`). After microelectronics normalization the vocabulary reached 6,677 entries (snapshot `component_canonical_vocab_v1.0.snapshot.json`, 2026-04-15), which is the state from which biotechnology and pharmaceuticals were normalized (2026-05-14). After both later pilots the vocabulary reached 10,856 entries (current `data/component_canonical_vocab_global_primary.json`). The two pilots therefore added roughly equivalent numbers of new entries (+3,843 during microelectronics, +4,179 during biotechnology and pharmaceuticals).

Per-domain pre-existing coverage at the time of normalization.

For each domain we computed the fraction of unique raw component names in the pilot CSVs whose normalized key was already present in the canonical vocabulary at the moment that domain's normalization first ran. The relevant snapshot was used for each domain (microelectronics against the pre-goldstandard snapshot; biotechnology and pharmaceuticals against the v1.0 snapshot).

Table 6: Pre-existing canonical-vocabulary coverage of unique raw component names per domain at the time of first normalization.

Domain	Vocab size at start	Unique raw components	Already in vocab	Coverage
Microelectronics	2,834	2,656	323	12.2%
Biotechnology	6,677	2,633	387	14.7%
Pharmaceuticals	6,677	2,594	374	14.4%

The three domains entered normalization with similar pre-existing canonical-vocabulary coverage of their incoming raw component names. Biotechnology and pharmaceuticals had slightly higher fractional coverage than microelectronics did at its start, because the v1.0 vocabulary, while dominated by microelectronics-specific

entries, also contained generic manufacturing-component canonical names (housings, fasteners, motors, sensors) that overlap with biotechnology and pharmaceutical instruments.

Per-technology first-touch cache hit rates.

A complementary measure aggregates the `cached_components_raw_count` field from the `normalization_manifest_*.json` produced at the earliest normalization timestamp per technology in the code repository: microelectronics 392 / 1,810 raw component-row occurrences cached at first touch (21.7%); biotechnology 613 / 1,531 (40.0%); pharmaceuticals 586 / 1,512 (38.8%). This per-technology measure counts each component once per technology, so it captures within-domain re-use of canonical names across technologies; biotechnology and pharmaceuticals score higher than microelectronics by this measure because their component vocabularies are more internally repetitive (many bioreactor instruments share a similar set of canonical parts).

Frozen-vocabulary re-normalization.

We re-normalized all 60 cross-domain raw CSVs (10 raw CSVs per (domain, configuration) combination, three domains, two configurations) against the post-pilot vocabulary using a `--freeze-vocab` flag added to the normalization script (`scripts/normalize_outputs_global_granularity.py` in the code repository). In freeze mode the normalizer skips the LLM call for unknown components and does not write any new entries to the vocabulary JSON. Outputs were written to `output/normalized_frozen_vocab/` in the code repository and analyzed by `scripts/cross_domain_stability_analysis_frozen_vocab.py` in the paper repository.

Table 7: Equivalence between live-vocabulary normalization (`output/normalized/`) and frozen-vocabulary re-normalization (`output/normalized_frozen_vocab/`) at the (technology, run) canonical-component-set level.

Domain-config	(tech, run) pairs	Identical sets	Differing sets	Total set symdiff
microelectronics $v1v1v1$	300	300	0	0
microelectronics $d3v1v1$	293	293	0	0
biotechnology $v1v1v1$	264	264	0	0
biotechnology $d3v1v1$	289	289	0	0
pharmaceuticals $v1v1v1$	241	241	0	0
pharmaceuticals $d3v1v1$	293	293	0	0

The frozen-vocabulary re-normalization produces canonical-component sets that are exactly identical to the live-normalization output at the level of every (technology, run) pair. The cross-domain Wilcoxon results are therefore unchanged: microelectronics median $\Delta = -0.173$, W=160, $p < 0.0001$; biotechnology $\Delta = +0.107$, W=498,

p=0.0056; pharmaceuticals $\Delta = +0.036$, W=478.5, p=0.0092; combined $\Delta = -0.031$, W=7,134, p=0.55.

The equivalence is structural rather than coincidental: the post-pilot vocabulary is the union of canonical mappings emitted by the live LLM normalization across all three pilots, so a frozen re-run becomes a deterministic lookup that returns the same canonical names the live runs produced. Two arguments bound the residual vocabulary-maturity confound. First, the three domains entered normalization with similar pre-existing coverage of their raw component names (Table 6), so no domain enjoyed a substantial canonical-entry head start over the others. Second, within-domain pairwise Jaccard depends only on whether the same raw component is mapped to the same canonical name across runs within that domain, a property the vocabulary lookup preserves regardless of the absolute canonical name chosen. The residual uncertainty the analysis does not bound is whether a counterfactual re-normalization that forced the LLM to re-emit canonical names under the full post-pilot canonical-examples context would shift within-domain Jaccard. That sensitivity is flagged for future replication.

S.15 Vocabulary-structure correlation test

This subsection records the per-technology vocabulary-structure correlation test that operationalizes the candidate mechanism for the domain-dependent debate effect reported in Section 2.3. The analysis script is `scripts/vocab_structure_correlation.py` in the paper repository, and consumes the raw and normalized CSVs in the code repository at `output/raw/` and `output/normalized/` together with the canonical-vocabulary JSON at `data/component_canonical_vocab_global_primary.json`.

Metrics.

For each of the 177 paired technologies (60 microelectronics, 59 biotechnology, 58 pharmaceuticals) we compute three vocabulary-structure metrics from the available 5 $v1v1v1$ plus 5 $d3v1v1$ runs per (technology, configuration):

- **Metric A: canonical-component cardinality.** The number of distinct canonical component names surfaced for the technology across the union of all 10 (5 $v1$ + 5 $d3$) normalized runs.
- **Metric B: raw-name cardinality.** The number of distinct raw (pre-normalization) component names surfaced for the technology across the union of all 10 raw runs.
- **Metric C: average synonym-set size.** For each canonical appearing for the technology, the number of distinct raw names whose `_norm_key` maps to that canonical in `component_canonical_vocab_global_primary.json`, averaged across the canonicals appearing for that technology.

The per-technology delta is the same paired metric used in Table 2: median pairwise post-normalization Jaccard at $d3$ minus the same at $v1$.

Hypothesis under test.

The mechanism proposed during the original drafting of Section 2.3 was that microelectronics has a larger or more ambiguous component vocabulary than biotechnology and pharmaceuticals, and that this drives the sign of the per-technology delta. The directional prediction is therefore a NEGATIVE Spearman rank correlation between each of Metrics A–C and the per-technology delta: larger or noisier vocabulary should predict a more negative delta.

Spearman rank correlation with bootstrap 95% CIs.

We compute Spearman ρ between each metric and the per-technology delta, both pooled across all 177 paired technologies and within each domain. For each ρ we report a percentile bootstrap 95% CI with 10,000 resamples (`numpy.random.default_rng(seed=42)`).

Table 8: Spearman rank correlation between per-technology vocabulary-structure metrics and the per-technology paired Jaccard delta ($d3 - v1$). Bootstrap 95% CIs from 10,000 percentile-bootstrap resamples (seed 42). The hypothesis "vocabulary cardinality drives the sign of the debate effect" predicts negative ρ in every row; the data do not support this prediction.

Metric	Scope	n	ρ	95% CI	p
Canonical-component cardinality	pooled	177	+0.127	(−0.027, +0.276)	0.0920
	microelectronics	60	+0.089	(−0.185, +0.364)	0.4967
	biotechnology	59	−0.060	(−0.323, +0.221)	0.6496
	pharmaceuticals	58	+0.081	(−0.187, +0.333)	0.5461
Raw-name cardinality	pooled	177	+0.049	(−0.098, +0.198)	0.5208
	microelectronics	60	+0.035	(−0.210, +0.285)	0.7880
	biotechnology	59	+0.079	(−0.193, +0.335)	0.5502
	pharmaceuticals	58	−0.003	(−0.287, +0.284)	0.9815
Average synonym-set size	pooled	177	−0.095	(−0.232, +0.046)	0.2103
	microelectronics	60	−0.130	(−0.390, +0.144)	0.3223
	biotechnology	59	+0.215	(−0.046, +0.454)	0.1021
	pharmaceuticals	58	+0.005	(−0.254, +0.265)	0.9725

Per-domain metric distributions.

For context, Table 9 reports the medians of each metric and of the per-technology delta within each domain. The three domains have nearly equivalent canonical-component cardinality and raw-name cardinality medians, despite producing per-technology deltas of opposite sign.

Table 9: Per-domain median values of the three vocabulary-structure metrics and the per-technology paired delta. The qualitative ordering that the cardinality hypothesis would require (microelectronics > biotechnology, pharmaceuticals on the metric) is not present in Metrics A–B and only weakly present in Metric C.

Domain	n	Metric A median	Metric B median	Metric C median	Delta median
microelectronics	60	18.0	52.5	2.65	−0.146
biotechnology	59	21.0	54.0	2.40	+0.105
pharmaceuticals	58	20.0	53.5	2.34	+0.060

Interpretation.

None of the three metrics yields a pooled $|\rho| > 0.15$, all pooled 95% bootstrap CIs straddle zero, and the signs are inconsistent with the hypothesis (positive where the hypothesis predicts negative for Metrics A and B; mixed within-domain signs for Metric C). Within-domain correlations have wide CIs that all straddle zero at $n \approx 60$ per domain. The per-domain metric distributions (Table 9) further undercut the qualitative basis of the hypothesis: microelectronics does not have a larger canonical-component vocabulary than biotechnology or pharmaceuticals at the per-technology level in this measurement (median 18 versus 21 and 20). We therefore treat the mechanism behind the cross-domain sign split as an open question. The cross-domain effect itself is unchanged: it is statistically significant in all three domains and survives the frozen-vocabulary sensitivity check (Supplementary A).

Optional scatter.

Figure 13 plots Metric A against the per-technology delta, colored by domain.

S.16 Light-prompt-engineered baseline robustness check

Motivation.

The naive baseline used in Table 1 uses a maximally generic prompt (“What are the primary manufacturing components of a {technology}? List each component on its own line.”) with the system prompt “You are a helpful assistant.” A reasonable concern is that this baseline understates what a single-shot LLM can do, and that a practitioner who invested a small amount of prompt-engineering effort could close much of the apparent gap between the naive prompt and the canonical-normalization rung, weakening the “normalization is the dominant lever” interpretation.

Engineered prompt.

To test this, we constructed a deliberately minimal prompt-engineered variant. The variant makes only three changes to the naive baseline: (i) a role-conditioned system prompt (“You are an expert in industrial supply chain analysis.”), (ii) a one-sentence exclusion rule, and (iii) a JSON output format. The full user prompt is: “List the primary physical hardware components of a {technology}, excluding raw materials,

software, and services. Return as a JSON array of component names." We deliberately omitted granularity calibration, few-shot examples, and any enumeration of the canonical vocabulary, because including these would conflate the test with the pipeline's full prompt-engineering effort rather than testing a "light" practitioner reference.

Setup.

Both baselines were run on the same four gold-standard technologies (Smartphone, Single-use bioreactor, Pharmaceutical Lyophilizer, Rotary tablet press), five iterations each, using `gpt-4.1-mini` for extraction, `gpt-4.1` for canonical normalization, and `gpt-4.1` for judging. We measured per-technology mean pairwise Jaccard across the five runs (raw and post-normalization) and aggregate precision and recall against the canonical-mapped gold standard.

Results.

Table 10 reports the comparison.

Table 10: Strawman naive prompt versus light prompt-engineered prompt on the four gold-standard technologies (five runs each). Stability is the macro-mean pairwise Jaccard across technologies. Precision and recall are aggregated over technologies against the canonical-mapped gold standard. The two baselines produce essentially indistinguishable stability at the raw level; the normalization gain is unchanged at roughly +0.63 absolute in both cases.

Configuration	Union	Precision	Recall	Stability
Strawman naive (raw)	168	0.216	0.977	0.179
Engineered (raw)	207	0.209	1.000	0.182
Strawman naive + canonical norm.	54	0.566	0.977	0.805
Engineered + canonical norm.	56	0.541	1.000	0.831

Interpretation.

At the raw level, the engineered prompt does not measurably improve run-to-run stability (0.179 versus 0.182, within the noise of n=4). After applying the canonical vocabulary, stability rises to 0.805 in the strawman case and 0.831 in the engineered case; the absolute normalization gain is +0.626 for the strawman prompt and +0.649 for the engineered prompt, essentially equal. Aggregate precision is also unchanged (0.216 versus 0.209 raw; 0.566 versus 0.541 normalized); recall improves marginally because the engineered prompt recovers one component the strawman missed (a Smartphone false negative). The headline finding from §2.2, that canonical normalization delivers the dominant stability gain, is therefore not an artifact of using a maximally weak prompt: light prompt engineering alone does not close the gap, and the gain attributable to the canonical vocabulary is essentially unchanged.

Limitations.

The test is restricted to the four gold-standard technologies and five runs each. A 60-technology version on the microelectronics benchmark would tighten the bootstrap confidence intervals around the engineered raw stability but is unlikely to change the qualitative conclusion: a single-pass prompt without an enumerated vocabulary cannot eliminate surface-form variation across runs, which is the specific failure mode the canonical vocabulary addresses.

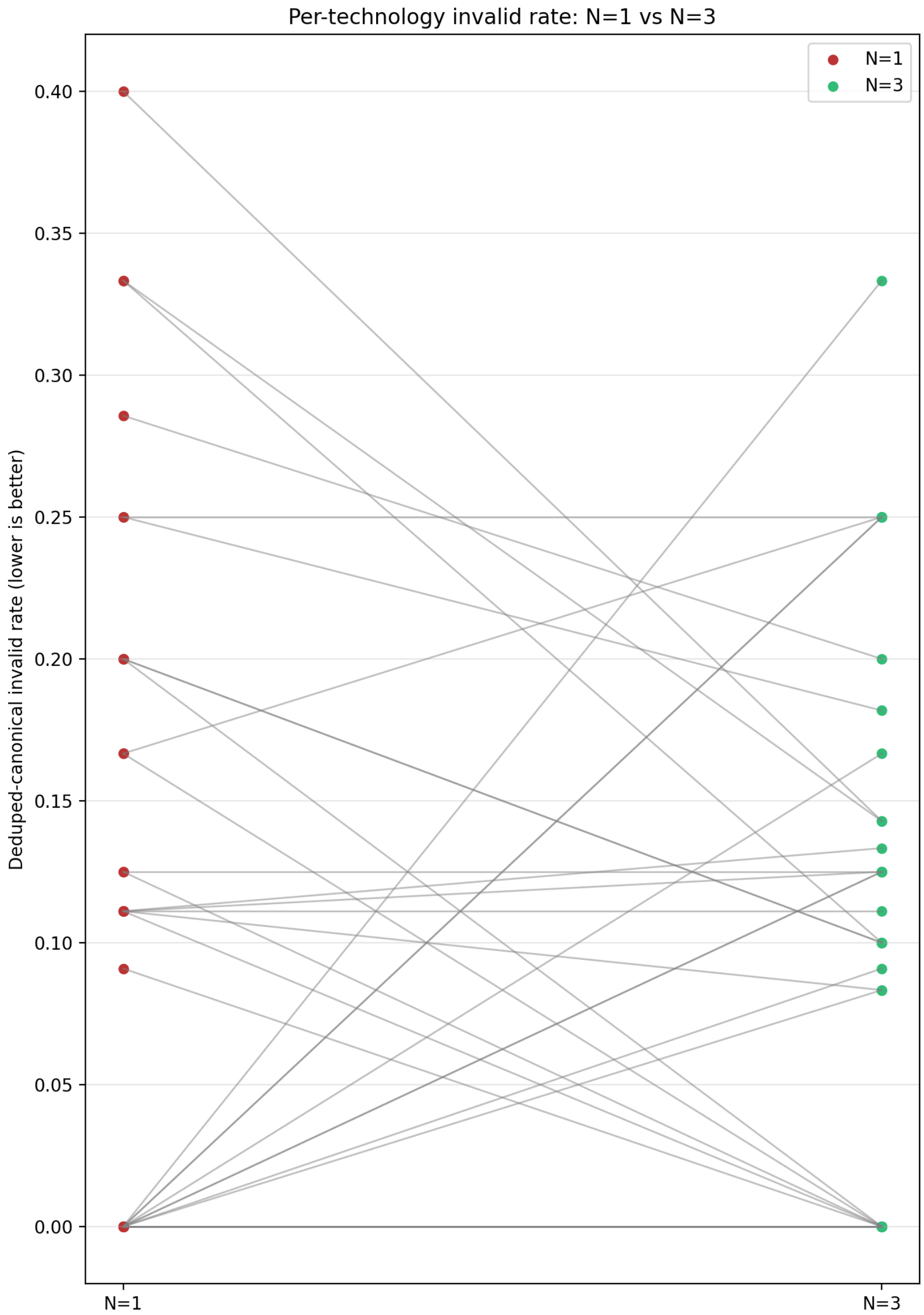

Fig. 8: **Per-technology paired comparison (N=1 vs N=3).** Each line corresponds to one technology, comparing deduped-canonical invalid rates under no debate (N=1) and moderate debate (N=3). This paired view highlights technology-to-technology variability and whether the typical technology improves under debate.

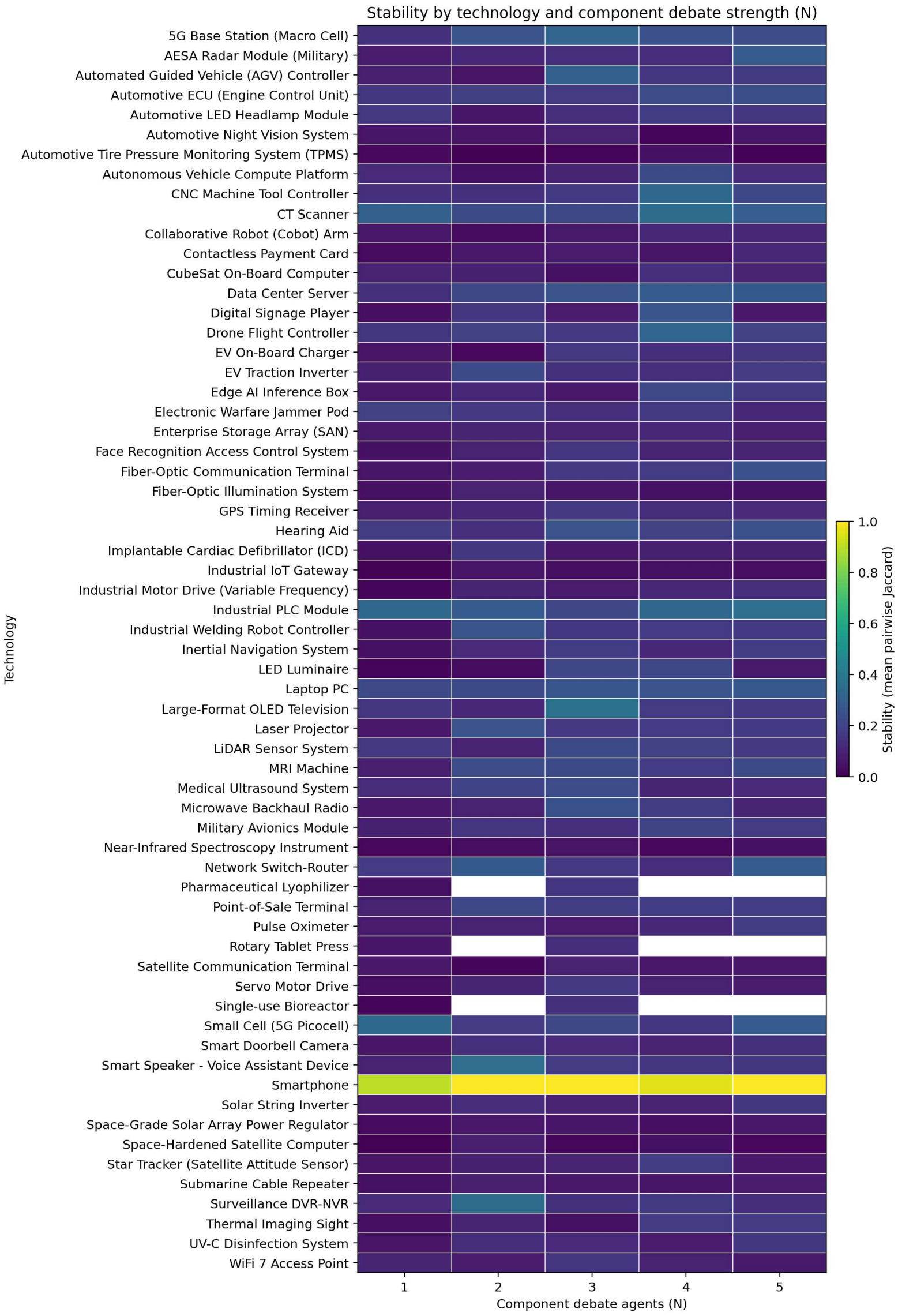

Fig. 9: **Robustness heterogeneity: stability heatmap by technology and debate strength.** Cell values show mean pairwise Jaccard similarity of Stage 1 final component sets across repeated runs for each technology and component debate strength N. This heatmap highlights that stability improvements under debate are heterogeneous across technologies, motivating macro-aggregated reporting and "sweet spot" selection.

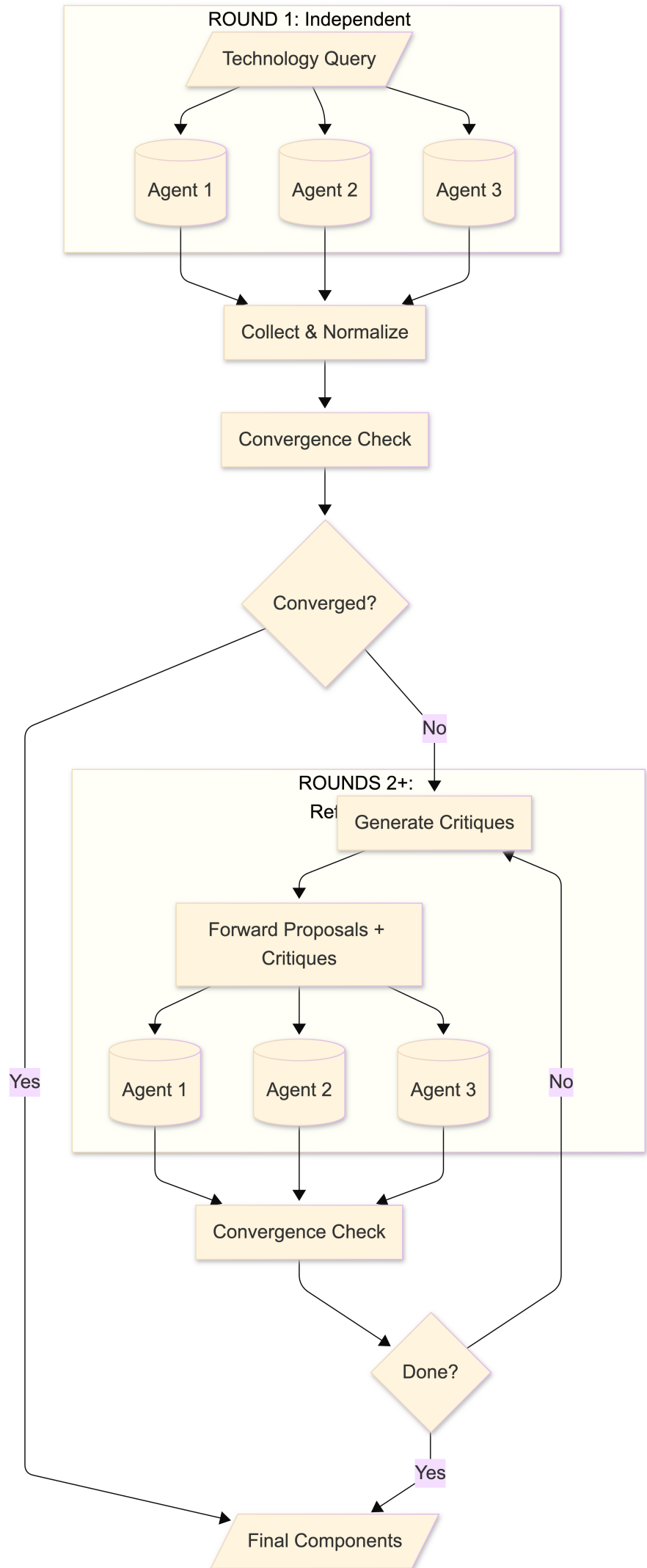

Fig. 10: Multi-agent debate within one extraction stage. In Round 0, three agents propose independently. If converged, debate ends; otherwise the system generates agreement feedback and refinement continues.

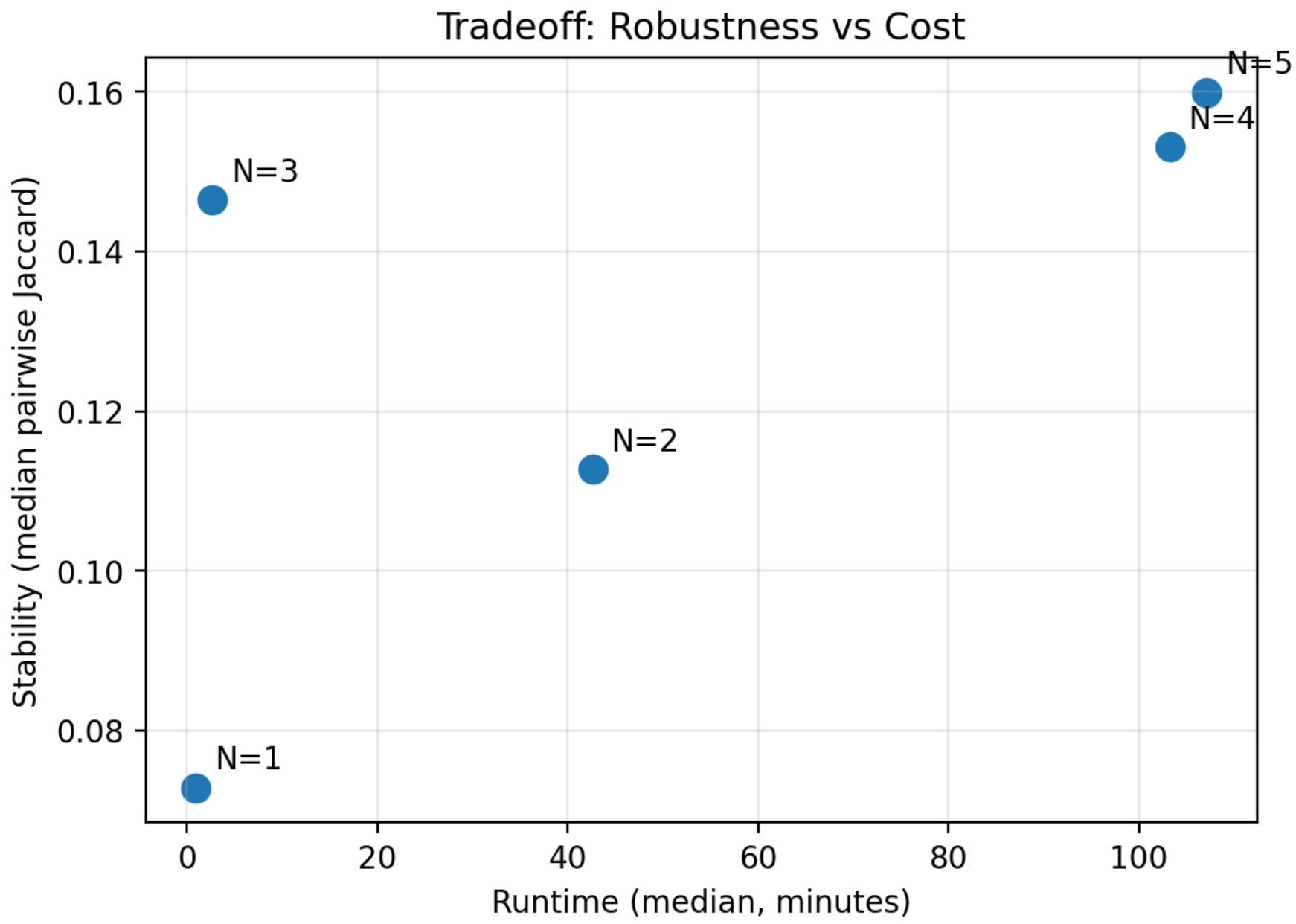

Fig. 11: Runtime versus stability trade-off across debate strengths $N \in \{1, 2, 3, 4, 5\}$, aggregated over 60 microelectronic technologies. Each point represents the macro-median; error bars show interquartile range across technologies. The cost of stronger debate grows roughly linearly with N while the marginal stability benefit shrinks past N=3, motivating the sweet-spot argument in Section 2.5.

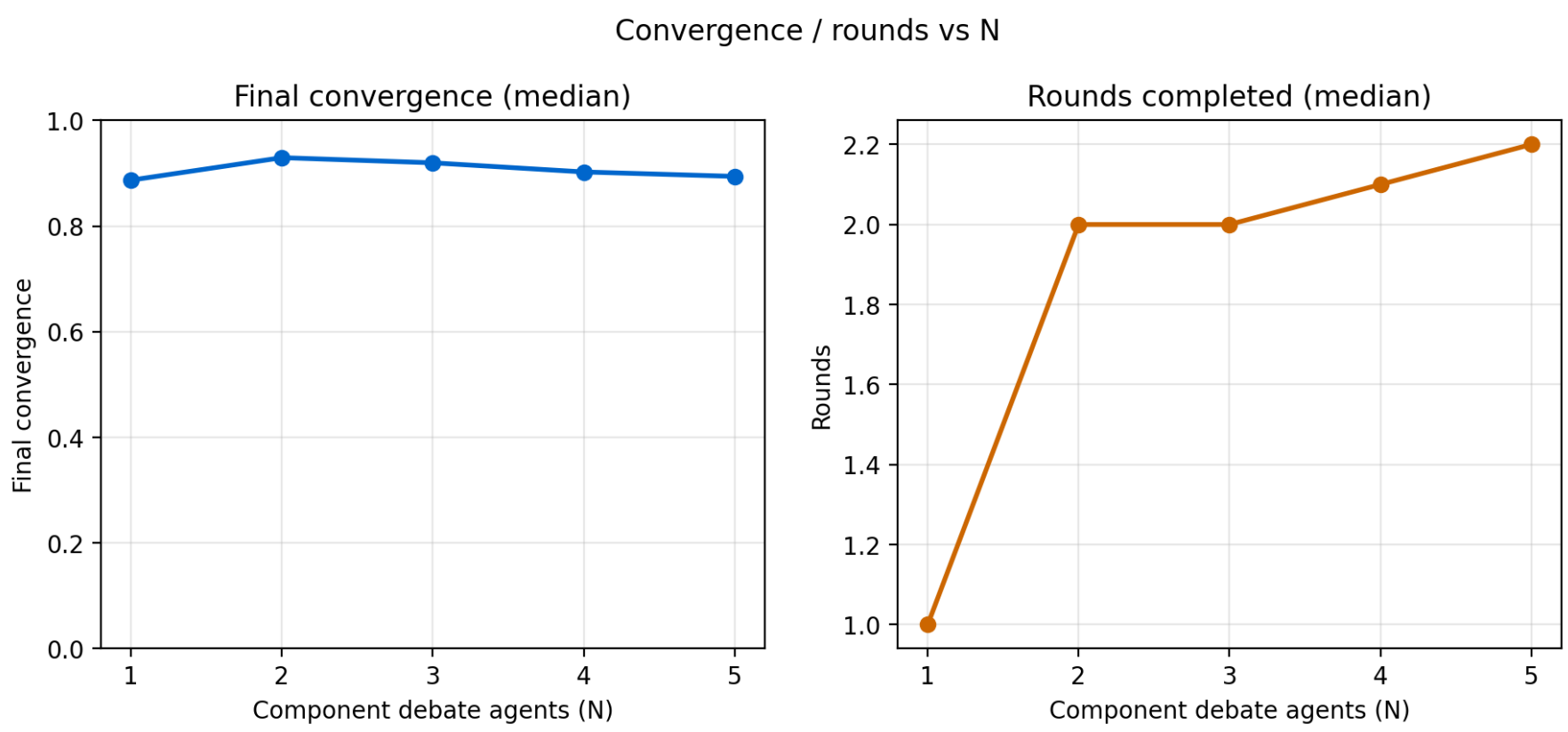

Fig. 12: Mean number of debate rounds to convergence as a function of debate strength N, aggregated over 60 microelectronic technologies. Stronger debates trigger more revision cycles on average, contributing to the runtime growth shown in Figure 11.

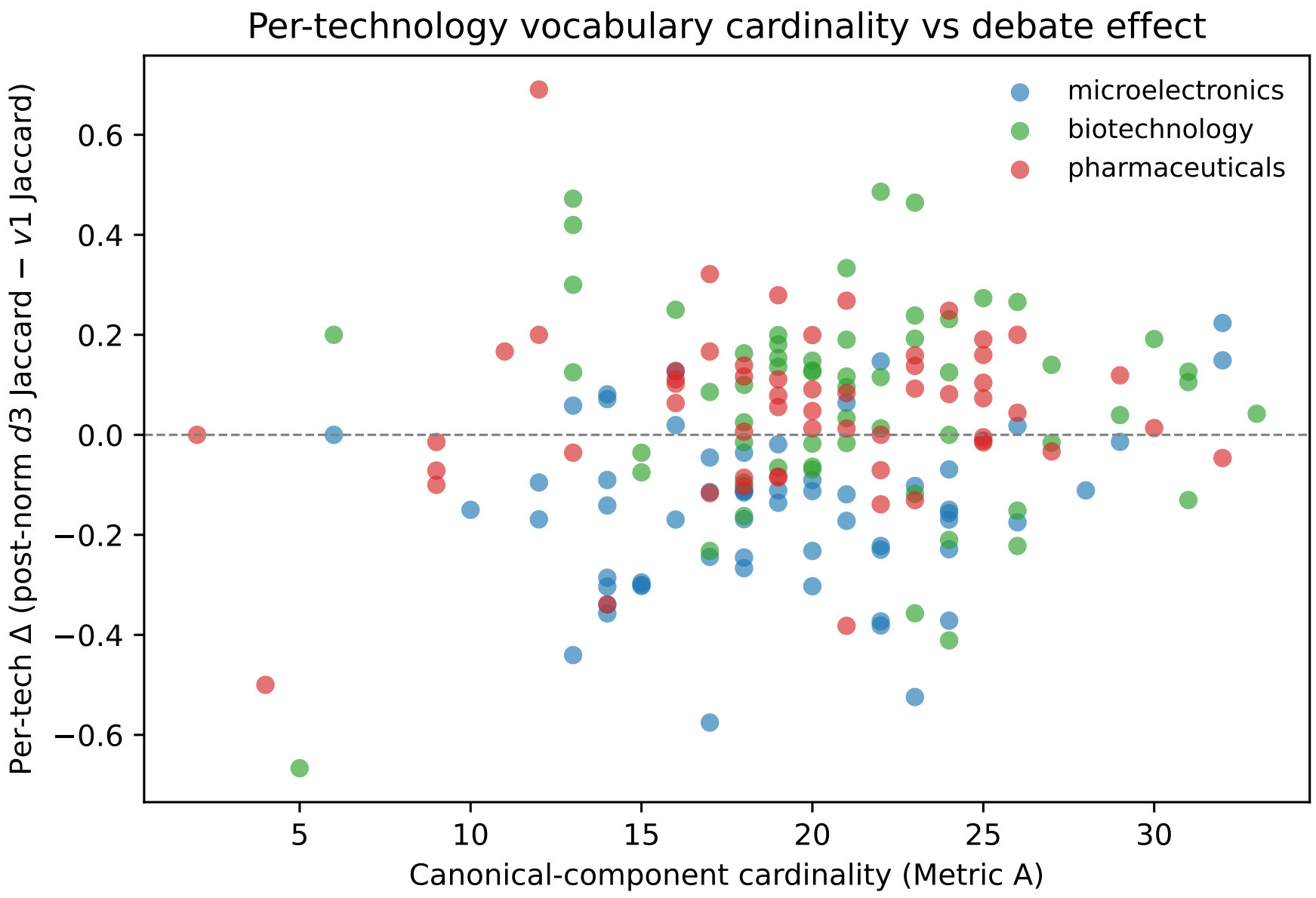

Fig. 13: Per-technology canonical-component cardinality (Metric A) versus per-technology paired delta ($d3 - v1$) in post-normalization Jaccard. Each point is one technology; colors distinguish the three domains. The horizontal dashed line marks $\Delta = 0$. The pooled and within-domain Spearman correlations between this metric and the delta are weak (Table 8).
